# Optimal solid state neurons

**DOI:** 10.1038/s41467-019-13177-3

**Published:** 2019-12-03

**Authors:** Kamal Abu-Hassan, Joseph D. Taylor, Paul G. Morris, Elisa Donati, Zuner A. Bortolotto, Giacomo Indiveri, Julian F. R. Paton, Alain Nogaret

**Affiliations:** 1 Department of Physics, University of Bath, Claverton Down, Bath, BA2 7AY UK; 2 School of Physiology, Pharmacology and Neuroscience, University of Bristol, Bristol, BS8 1TD UK; 3 Institute of Neuroinformatics, University of Zürich and ETH Zürich, Winterthurerstrasse 190, 8057 Zürich, Switzerland; 4 Department of Physiology, Faculty of Medical and Health Sciences, University of Auckland, Grafton, Auckland, New Zealand

**Keywords:** Biomedical engineering, Electrical and electronic engineering, Electronics, photonics and device physics

## Abstract

Bioelectronic medicine is driving the need for neuromorphic microcircuits that integrate raw nervous stimuli and respond identically to biological neurons. However, designing such circuits remains a challenge. Here we estimate the parameters of highly nonlinear conductance models and derive the ab initio equations of intracellular currents and membrane voltages embodied in analog solid-state electronics. By configuring individual ion channels of solid-state neurons with parameters estimated from large-scale assimilation of electrophysiological recordings, we successfully transfer the complete dynamics of hippocampal and respiratory neurons in silico. The solid-state neurons are found to respond nearly identically to biological neurons under stimulation by a wide range of current injection protocols. The optimization of nonlinear models demonstrates a powerful method for programming analog electronic circuits. This approach offers a route for repairing diseased biocircuits and emulating their function with biomedical implants that can adapt to biofeedback.

## Abstract (web summary)

Designing efficient and scalable specialized neuromorphic circuits to integrate raw nervous stimuli and respond identically to biological neurons remains a challenge. Here, the authors propose an analog programming strategy to emulate biological neurons in silico.

## Introduction

The electrical properties of biological cells have long been studied to understand the intracellular dynamics underpinning membrane voltage oscillations^1^. The difficulty of measuring microscopic parameters that control the dynamics of ionic currents^2^ and the nonlinearity of ionic conductances^3^ has hampered so far theoretical efforts to build quantitative computational models and subsequently neuromorphic devices replicating the exact response of a biological neuron. Although silicon neurons^4–8^, synapses^9^ and brain inspired networks^10–16^ have been proposed, these designs were not meant to reiterate the behaviour of biological cells in complete detail, but to search for the organizing principles of biology that can be applied to practical devices. The increasing focus on implantable bioelectronics to treat chronic disease is however changing this paradigm and is instilling new urgency in the need for low-power analogue solid-state devices that accurately mimic biocircuits. Analogue asynchronous electronics is the most promising way to integrate raw nervous stimuli^17^ instantaneously, independently of system size and complexity. Recent efforts at building quantitative computational models of neurons have focussed on generalizing the Hodgkin–Huxley (HH) model to multi-channel models^18,19^. Approaches ranging from hand-tuning^20^ to trial-and-error fitting^21–24^, multi-objective functions^25–28^, genetic algorithms^29^, Bayesian inference^30^ and statistical interpolation^31–34^ have been implemented to estimate maximal ionic conductances. Constrained nonlinear optimization has further allowed nonlinear parameters, such as voltage thresholds and recovery times to be inferred, which was key to predicting the dynamic state of a biological neuron^35–38^. Transferring dynamic information from a biological cell to a biomimetic circuit is met with additional difficulties arising from hardware constraints^39–42^. For example, conventional silicon technology^4,43^ assigns a constant thermal voltage to the width of the transition region from the open to the closed state of an ionic gate. In biology, this width varies from one type of neuron to another. A biologically accurate neuromorphic design is therefore needed, whose mathematical model is compatible with nonlinear optimization, and that responds identically to a biological neuron under any current injection.

Here, we propose an analogue circuit modelling generic ion channels designed for this purpose. Ab initio analysis of a solid-state neuron (SSN) implementing these ion channels gives the equations that describe the rate of change of the membrane voltage and gate variables. The SSN equations have a form similar to the HH model, yet derive from an analogue circuit with transistors operated in the weak inversion (or sub-threshold) domain^44^, which are most relevant to making low-power bioimplants. The specific activation curves and gate kinetics of individual ion channels are synthesized in silico through analogue interpolation^45,46^. We demonstrate the high fidelity of the analytical model to the electronics by observing nearly identical membrane voltage oscillations in response to the same current injection protocols. This gave a high degree of confidence that bias parameters extracted from model optimization could be automatically dialled in the electronic device to predict biological behaviour. The equivalence between the SSN model and SSN hardware was further confirmed by twin experiments, which recovered the parameter configuration of the hardware by assimilating its membrane voltage with the SSN model. A three-ion channel SSN model incorporating the transient sodium, non-inactivating potassium and leakage channels (NaKLs) was constructed and found to predict the spike timings of the HH model with 96.4% accuracy. Finally, we built six-channel silicon devices that faithfully model CA1 hippocampal and respiratory neurons. The completed models predict the membrane voltage of biological neurons in excellent agreement (94–97%) with the membrane voltage oscillations observed in response to 60 different current protocols. We also discuss the dynamics of gate variables and ionic currents predicted by the SSN model. These results demonstrate the possibility of making bionic chips that can reproduce the response of biological cells in terms of electrical activity.

## Results

### Twin experiment with an SSN

**Fig. 1:**
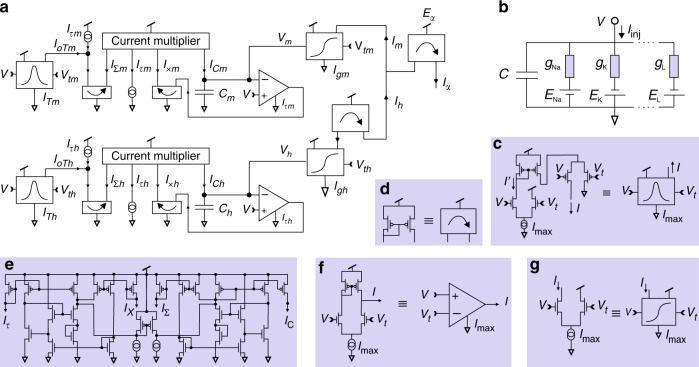
Biomimetic solid-state ion channel. **a** The conductance of ion species $\alpha$ is modulated by an activation gate and an inactivation gate. The net ionic current, ${I}_{\alpha }=\ ({I}_{m}-{I}_{h})\theta ({I}_{m}-{I}_{h})$, is the difference between the activation current (${I}_{m}$) and the inactivation current (${I}_{h}$). The Heaviside function, $\theta ()$, specifies that the current mirror outputs a positive current ${I}_{\alpha }$ when ${I}_{m}\, > \, {I}_{h}$ and 0 otherwise. **b** Electrical equivalent circuit of the neuron membrane. **c**–**g** Block diagrams of sub-circuits for **c** the gate recovery time, **d** current mirror, **e** current multiplication ${I}_{C\gamma }={I}_{\times \!\gamma }\times {I}_{\tau \gamma }/{I}_{\Sigma \gamma }$, where $\gamma \in \left\{m,h\right\}$, **f** transconductance amplification and **g** sigmoidal activation/inactivation.

**Fig. 2:**
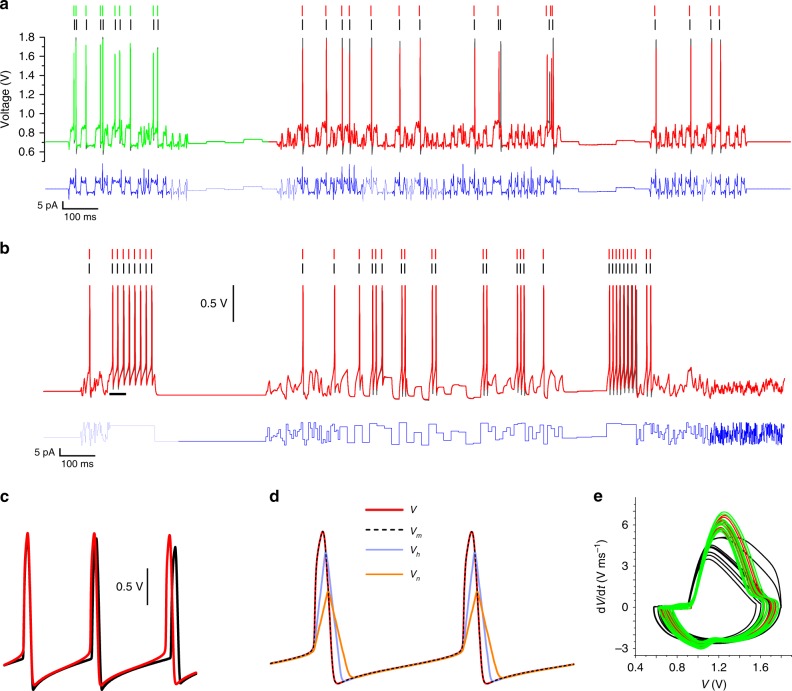
Twin experiment with a solid-state neuron. **a** Membrane voltage of a sub-threshold neuron (black line) stimulated by a current protocol mixing hyperchaotic oscillations with current steps (blue line). The membrane voltage was measured from a VLSI chip implementing the NaKL SSN model. The SSN model was synchronized to the data over a *T* = 600-ms-long assimilation window (green line). The complete model configured with the estimated parameters was then forward integrated from *t* = 600 ms onwards to *t* = 2000 ms to predict the membrane voltage (red line). **b** Membrane voltage predicted by the completed SSN model for a different current protocol consisting of fast and slow-varying steps (red line). VLSI membrane voltage measured on the VLSI neuron (black line). **c** Detail of membrane voltage oscillations showing the shape of predicted and actual action potentials. **d** Predicted time dependence of state variables $V$, ${V}_{m}$, ${V}_{h}$ and ${V}_{n}$. **e** Phase portrait of action potentials over the assimilation window: VLSI experiment (black line), fitted (green line) and predicted from *t* = 0 (red line).

The SSN model is first validated by its ability to predict the membrane voltage oscillations of the SSN hardware implemented in VLSI (Fig. 1) when both are configured with the same parameters and stimulated with the same current protocol. The comparison between the NaKL very-large-scale integration (VLSI) hardware and the SSN model biassed with the “VLSI” parameters of Table 1 is shown in Supplementary Fig. 1. The quantitative agreement between model and experiment is remarkable and validates the SSN model to within experimental error. This excellent agreement calls for one further test consisting in estimating the parameter configuration of the silicon chip by assimilating the membrane voltage oscillations of the silicon chip with the SSN model. In this way, one seeks to perform a “twin experiment” to recover the chip parameters, the assumption being that if the model is inaccurate, the extracted parameters will necessarily differ from those set in the chip. We used a 600-ms-long assimilation window to synchronize the SSN model to the observed membrane voltage (Fig. 2a, black line). The optimum fit of the model to the data is shown as the green line (Fig. 2a) and the extracted parameters are shown in the column “VLSI $\to$ SSN” in Table 1. These parameters are in good agreement with the original parameters (“VLSI” column). Well-constrained parameters such as ${\tilde{I}}_{\tau m}$ or $\alpha$ are estimated to be within 0–4% of the value set in VLSI. Parameters that are less well constrained exhibit greater uncertainty on their estimates. This uncertainty is due to the assimilation of experimental error embedded in measurements of the VLSI membrane voltage. Residual error in the data introduces uncertainty in the parameter field, which is absent from twin experiments assimilating clean model data^36^. Within experimental error, the good agreement between initial and estimated parameters suggests that Eq. (10) is a highly accurate model of the VLSI hardware.

**Table 1:** Parameters estimates for the NaKL SSN model.

Ion	Parameter		VLSI	Lower bound	Upper bound	VLSI → SSN	HH → SSN
NaT	${\tilde{I}}_{gm}$ (nA pF^−1^)		5	0	200	9.70	162.75
	${V}_{tm}$ (V)		0.9	0.01	1.8	1.011	0.908
	${\beta }_{m}$ (V^−1^)		13	1	100	15.37	8.405
	${\tilde{I}}_{\tau m}$ (nA pF^−1^)		100	0.1	200	100	0.6854
	${\tilde{I}}_{gh}$ (nA pF^−1^)		5	0	200	8.924	4.638
	${V}_{th}$ (V)		0.9	0.01	1.8	1.004	1.143
	${\beta }_{h}$ (V^−1^)		13	1	100	16	3.581
	${\tilde{I}}_{\tau h}$ (nA pF^−1^)		0.33	0.1	200	1.1	0.1482
K	${\tilde{I}}_{gn}$ (nA pF^−1^)		2.5	0	200	3.303	164.18
	${V}_{tn}$ (V)		1.1	0.01	1.8	1.20	0.911
	${\beta }_{n}$ (V^−1^)		13	1	100	14.50	8.372
	${\tilde{I}}_{\tau n}$ (nA pF^−1^)		0.4	0.1	200	0.54	0.6747
Leak	${\tilde{I}}_{\mathrm{L}}$ (nA pF^−1^)		0.1	0	100	0.1195	0.23105
	${\beta }_{\mathrm{L}}$ (V^−1^)		13	1	100	11.132	1
	${E}_{\mathrm{L}}$ (V)		0.7	0.001	1.8	0.6949	0.6194
	$\alpha$		1	10^−4^	1000	0.96	39.54
	$\beta$ (V^−1^)		13	10	16	16	14
	${\tilde{I}}_{\mathrm{dark}}$ (nA pF^−1^)			−0.05	+0.05	+0.009	0

Column 3 lists the voltage thresholds, current biases and sigmoidal parameters, which are set in the VLSI micro-circuit implementing the SSN model in silico. Columns 4 and 5 specify the parameter search intervals used in data assimilation. Column 6 lists the SSN parameters inferred by assimilating the membrane voltage of the VLSI neuron (twin experiment). These parameters ought to be the same as the VLSI parameters (column 3). Column 7 gives the SSN parameters estimated by assimilating the membrane voltage synthesized by the Hodgkin–Huxley (HH) model (Supplementary Table 1). ${I}_{\mathrm{inj}}$ had units of nA, and $V$, ${V}_{m}$, ${V}_{h}$ and ${V}_{n}$ units of V

The same SSN model configured with the “VLSI → SSN” parameters gave excellent predictions of the VLSI membrane voltage. The completed model was forward integrated from the end of the assimilation window (Fig. 2a, red trace) and from the start of another epoch stimulated with a different current protocol (Fig. 2b). Details of the observed and predicted action potentials show that the model correctly replicates the shape of spikes. Small phase slips occur in the predicted oscillations (Fig. 2c), which self-correct over the epoch duration. The time dependences of gate variables ${V}_{m}$, ${V}_{h}$ and ${V}_{n}$ in the SSN model are compared to the membrane voltage $V$ (Fig. 2d). One notes the large delay of $K$ activation (${V}_{n}$) relative to Na inactivation (${V}_{h}$), which is itself slower that Na activation (${V}_{m}$). ${V}_{m}$ is quasi-synchronous with membrane depolarization ($V$). This activation/inactivation sequence is consistent with the sequence observed in most biological neurons^46^. Phase portraits of action potentials (Fig. 2e) show good agreement between SSN model and experiment, except at the onset of depolarization.

### Assimilation of HH model data

**Fig. 3:**
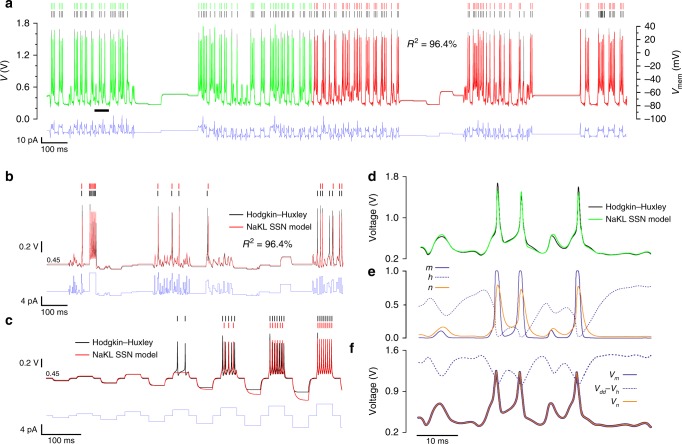
Equivalence of the NaKL SSN model and the Hodgkin–Huxley model. **a** The equivalence between the two models is demonstrated by synchronizing the SSN model to membrane voltage oscillations synthesized by the Hodgkin–Huxley model (black line). The Hodgkin–Huxley time series voltage was assimilated over a 1000-ms-long window (green line) under the constraints of the current injection protocol (blue line). The membrane voltage was predicted from *t* = 1000 ms onwards by forward integrating the current protocol with the completed SSN model (red line). **b**, **c** Membrane voltages predicted by the same SSN model (red line) and HH model (black line) for two different current protocols. **d** Detail of the SSN and HH action potentials and a comparison of NaT and K gate variables in **e** the HH model and **f** the SSN model.

Next, we demonstrate the equivalence of the NaKL SSN model (Eq. (10)) and the HH model (Eq. (11)) by predicting the state of the HH neuron with the NaKL SSN model. This approach has two merits: first to validate the dynamics of gate variables (${V}_{\gamma }$), which are not accessible to observation in biological cells, but are given by the HH model, and second to assess the fidelity of information transfer from one NaKL model to another. An HH model was initially used to compute the membrane voltage time series (Fig. 3a, black lines) by forward integrating a complex current protocol (blue traces). This model used the parameters of a thalamic relay neuron listed in Supplementary Table 1. The SSN model was then synchronized to the HH membrane voltage over a 1000-ms-long assimilation window (green trace) and gave the parameter estimates listed in Table 1 (“HH → SSN” column). These parameters are used to configure the complete SSN model. The complete model was then forward integrated from the end of the assimilation window onwards (Fig. 3a, red trace), and in two novel current protocols (Fig. 3b, c). The state of the neuron calculated at the end of the assimilation window provided the initial conditions of forward integration. In Fig. 3b, c the initial state of the neuron was the rest state: $V={V}_{m}={V}_{h}={V}_{n}=0.466$ V. The spike coincidence factor^47^ between HH and SSN models was $\Gamma$ = 97% in Fig. 3a and 91% in Fig. 3b. The metric used to quantify the match between HH and SSN voltage time series was ${R}^{2}=1-{\mathrm{NRMSD}}$, where NRMSD is root mean square deviation between data and predictions normalized by the amplitude of membrane voltage oscillations (1.8 V). We found ${R}^{2}=96.4 \%$. Discrepancies between SSN predictions and HH data are mainly observed during extreme hyperpolarizing current steps (Fig. 3c). This occurs because the relatively weak hyperpolarizing currents used in the assimilation protocol fail to fully constrain sub-threshold parameters.

The parameters estimated from the HH data (Table 1, “HH → SSN” column) give high maximum ion currents ${I}_{g\gamma }$, $\gamma \in \left\{m,h,n\right\}$. These currents are about 40 times larger than currents estimated from the VLSI chip, in spite of current injection being of similar magnitude. Current injection is also re-scaled by $\alpha =39.54$. This re-scaling of currents is a consequence of re-scaling the membrane voltage from the [−100, +45 mV] range of the HH model to the [0, 1.8 V] range. Parameter $\alpha$ also accounts for the actual neuron surface area—typically ${\mathrm{ISA}}=2.9\times 1{0}^{-4}$ cm^2^ ^46^—that absorbs the injected current ${J}_{\mathrm{inj}}={I}_{\mathrm{inj}}/{\mathrm{ISA}}$ in the HH model and biological neurons (Eq. (11)). These simple considerations give $\alpha \sim (12.414/{\mathrm{ISA}}+1241.4)/1000\approx 44$, which agrees with the optimum value of $\alpha =39.54$ (Table 1). The estimated voltage thresholds ${V}_{{t}\gamma }$ are consistent with the relative positions of known biological thresholds (Supplementary Table 1). The estimated ${\tilde{I}}_{\tau \gamma }$ give recovery times: ${t}_{0,m}={\mathrm{U}}_{T}/{\tilde{I}}_{{\tau }_{m}}=0.026/0.6854=0.03$ ms, ${t}_{0,h}=0.026/0.1482=0.17$ ms and ${t}_{0,n}=0.026/0.6747=0.04$ ms.

Figure 3d shows that the action potentials of the SSN model (green line) and HH model (black line) are nearly identical. The dynamics of HH gate variables $m$, $h$ and $n$ is shown in Fig. 3e together with the same for SSN gate variables ${V}_{m}$, ${V}_{h}$ and ${V}_{n}$ (Fig. 3f). These plots demonstrate the closeness of the HH and SSN model. The gate variables ${V}_{m}$ and ${V}_{n}$ have greater correlation than $m$ and $n$ because the former are only delayed relative to the membrane voltage, whereas latter include both retardation and threshold.

We now have a systematic methodology for transferring information from a biological neuron to an SSN neuron. Because mammalian neurons are more complex than the NaKL model (Table 2), one expands Eq. (10) to include these extra ionic currents.

**Table 2:** Ionic currents of hippocampal (CA1) and RN.

ID	Channel	Current density	CA1	RN
NaT	Transient Na^+^	${J}_{\mathrm{NaT}}={g}_{\mathrm{NaT}}{m}^{3}h({E}_{\mathrm{Na}} - V)$	Yes	Yes
NaP	Persistent Na^+^	${J}_{\mathrm{NaP}}={g}_{\mathrm{NaP}}m({E}_{\mathrm{Na}}-V)$	Yes	Yes
K	Non-inactivating K^+^	${J}_{\mathrm{K}}={g}_{\mathrm{K}}{m}^{4}({E}_{\mathrm{K}}-V)$	Yes	Yes
A	Rapidly inactivating K^+^	${J}_{{\mathrm{K}}_{\mathrm{A}}}={g}_{{\mathrm{K}}_{\mathrm{A}}}{m}^{4}h({E}_{\mathrm{K}}-V)$	Yes	Yes
AHP	Calcium-activated K^+^	${J}_{{K}_{\mathrm{AHP}}}={g}_{{\mathrm{K}}_{\mathrm{AHP}}}m({E}_{\mathrm{K}}-V)$	d.d.	Rare
CaL	High threshold Ca^2+^	${J}_{\mathrm{CaL}}=\rho {m}^{2}{J}_{\mathrm{Ca}}$	d.d.	Rare
CaT	Low threshold Ca^2+^	${J}_{\mathrm{CaT}}={m}^{2}h{J}_{\mathrm{Ca}}$	d.d.	Rare
HCN	Hyperpolarisation-activated cation	${J}_{\mathrm{HCN}}={g}_{\mathrm{HCN}}h({E}_{\mathrm{HCN}}-V)$	d.d.	d.d.
M	Muscarinic-sensitive K^+^	${J}_{\mathrm{M}}={g}_{\mathrm{M}}m({E}_{\mathrm{K}}-V)$	Yes	No
Leak	Leak channels	${J}_{\mathrm{L}}={g}_{\mathrm{L}}({E}_{\mathrm{L}}-V)$	Yes	Yes

*RN* respiratory neuron, *d.d.* distal dendrite

Ion current densities ${J}_{\alpha }$ of conductance models as a function of ionic conductances ${g}_{\alpha }$, reversal potentials ${E}_{\mathrm{Na}}\approx +45$ mV, ${E}_{\mathrm{K}}\approx-90$ mV, ${E}_{\mathrm{HCN}}=-43$ mV^70^ and maximum calcium current ${J}_{\mathrm{Ca}}$ The ionic currents of the solid-state model ${I}_{\alpha }=\ ({I}_{m}-{I}_{h})\theta ({I}_{m}-{I}_{h})$ are given by Eq. (10). Prevalence of ion channels in CA1 neurons^48^ and respiratory neurons^55,56^ distinguishing soma and distal dendrites (d.d.)

### SSN model of a CA1 pyramidal cell from the rat hippocampus

The model of the CA1 neuron included the ion channels present in high density in the soma and proximal dendrites^48^: the transient Na^+^ current (NaT) that initiates action potentials, the depolarization activated K^+^ current (K) and the A-type K^+^ current that repolarizes the membrane after a delay. Long-lived persistent Na^+^ current (NaP) and muscarinic-sensitive K^+^ current (M) were included for their known contribution to bursting dynamics^49,50^. However, both the low threshold calcium current (CaT)^51,52^ and the hyperpolarisation-activated cation current (HCN)^53^ were omitted as these ion channels are mainly located in apical dendrites. Their density is low in the soma compartment in which current is injected. The after-hyperpolarization (AHP) current may be observed in CA1 neurons under voltage clamp conditions^54^; however, it gives a residual contribution to neuron adaptation in current-clamp experiments^54^. Therefore, the AHP current was also omitted from the model. This assumption is supported by our observation that SSN models that include the AHP channel are unable to predict membrane voltage oscillations, whereas those including the M-channel instead give good predictions. The CA1 SSN model was thus expanded to include the NaP, A and M currents alongside the NaT, K and Leak, giving eight coupled differential equations in total.

**Fig. 4:**
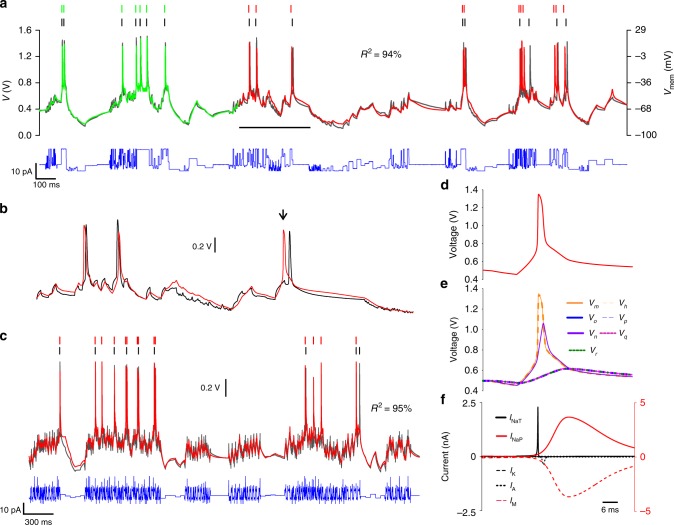
Assimilation and prediction of a CA1 pyramidal neuron. Membrane voltage oscillations of a pyramidal cell from the rat hippocampal cortex (black line) induced by the injection of a current protocol (blue line). The current trace shows the actual injected current, as measured. The CA1 SSN model was synchronized to the experimental membrane voltage over a *T* = 940-ms-long assimilation window (green trace). The optimum fit produced an estimate of the model parameters shown in Table 3. Models completed by incorporating the optimal parameters were used to predict the membrane voltage from $t\ge T$ (red line). **b** Detail of the predicted membrane voltage over the time interval indicated by the horizontal bar. **c** Further predictions were made for a wide range of current protocols one of which is shown here. Detailed dynamics of state variables of the SSN model during an action potential: **d** membrane voltage, **e** gate variables and **f** ionic currents.

Figure 4a shows the best fit of this model (green line) to the CA1 neuron (black line) over a 940-ms-long assimilation window. The estimated parameters are listed in Table 3 (CA1 $\to$ SSN). The membrane voltage was then predicted from the end of the assimilation window over the next 1960 ms by integrating the current protocol (blue line) with the completed CA1 SSN model (red line). The spike coincidence factor was $\Gamma =29 \%$ and the agreement between predicted and observed oscillations was ${R}^{2}=94 \%$. A comparison of individual depolarization events and sub-threshold oscillations is made in Fig. 4b. The predictive power of the CA1 SSN model was tested on many other current protocols such as the one in Fig. 4c ($\Gamma =43 \%$, ${R}^{2}=95.5 \%$). Within this model an action potential (Fig. 4d) is initiated by the fast activation of the NaT channel (${V}_{m}$) followed by the slower activation of the K current (${V}_{n}$) (Fig. 4e). Data assimilation assigns the slowest activation to the M-type current (${V}_{r}$), A-type current (${V}_{o}$) and NaP current (${V}_{q}$). These currents are known to be long lived in CA1 cells where they support burst oscillations. In contrast the NaT activation gate (${V}_{m}$) has the shortest recovery time and initiates depolarization, while the K current is delayed to drive repolarisation. Therefore, data assimilation assigns kinetic parameters consistent with the known biological properties of ion channels. The predicted amplitudes of the NaT and K currents are in the ratio of 6.2:1 (Fig. 4f). This is also in excellent agreement with the 5.8:1 ratio of conductances used by Golomb et al.^48^ to model CA1 neurons. Hence, the CA1 SSN model had very good predictive power for the membrane voltage and inferred correctly most thresholds and kinetics parameters.

**Table 3:** SSN parameters extracted from biological neurons.

Ion	Parameter		CA1 → SSN	RN → SSN
NaT	${\tilde{I}}_{gm}$ (nA pF^−1^)		20	20
Activation	${V}_{tm}$ (V)		0.867	1.0311
	${\beta }_{m}$ (V^−1^)		3.307	10.498
	${\tilde{I}}_{\tau m}$ (nA pF^−1^)		20	0.3877
Inactivation	${\tilde{I}}_{gh}$ (nA pF^−1^)		19.95	0.665
	${V}_{th}$ (V)		0.866	0.8033
	${\beta }_{h}$ (V^−1^)		3.329	13.74
	${\tilde{I}}_{\tau h}$ (nA pF^−1^)		1.999	0.1
NaP	${\tilde{I}}_{gq}$ (nA pF^−1^)		5	0.4640
Activation	${V}_{tq}$ (V)		0.593	0.81714
	${\beta }_{q}$ (V^−1^)		21.711	31.443
	${\tilde{I}}_{\tau q}$ (nA pF^−1^)		0.01	20
K	${\tilde{I}}_{gn}$ (nA pF^−1^)		1.0	19.84
Activation	${V}_{tn}$ (V)		1.087	1.0323
	${\beta }_{n}$ (V^−1^)		10.0	10.587
	${\tilde{I}}_{\tau n}$ (nA pF^−1^)		0.154	0.3749
A	${\tilde{I}}_{go}$ (nA pF^−1^)		0.131	19.99
Activation	${V}_{to}$ (V)		0.629	1.0801
	${\beta }_{o}$ (V^−1^)		75.89	4.905
	${\tilde{I}}_{\tau o}$ (nA pF^−1^)		0.01	0.1017
Inactivation	${\tilde{I}}_{gp}$ (nA pF^−1^)		0.333	19.98
	${V}_{tp}$ (V)		0.885	1.0521
	${\beta }_{p}$ (V^−1^)		1.0	5.378
	${\tilde{I}}_{\tau p}$ (nA pF^−1^)		0.01	0.1
M	${\tilde{I}}_{gr}$ (nA pF^−1^)		5.0	0
Activation	${V}_{tr}$ (V)		0.593	
	${\beta }_{r}$ (V^−1^)		21.765	
	${\tilde{I}}_{\tau r}$ (nA pF^−1^)		0.01	
Leak	${\tilde{I}}_{\mathrm{L}}$ (nA pF^−1^)		0.66	0.1
	${\beta }_{\mathrm{L}}$ (V^−1^)		0.1	0.6059
	${E}_{\mathrm{L}}$ (V)		0.2	0.6753
	$\alpha$		1.586	7.795
	$\beta$ (mV^−1^)		10.384	10

Parameters extracted from a pyramidal neuron (CA1 $\to$ SSN) and from a respiratory neuron (RN $\to$ SSN)

### SSN model of a respiratory neuron from the rat brain stem

**Fig. 5:**
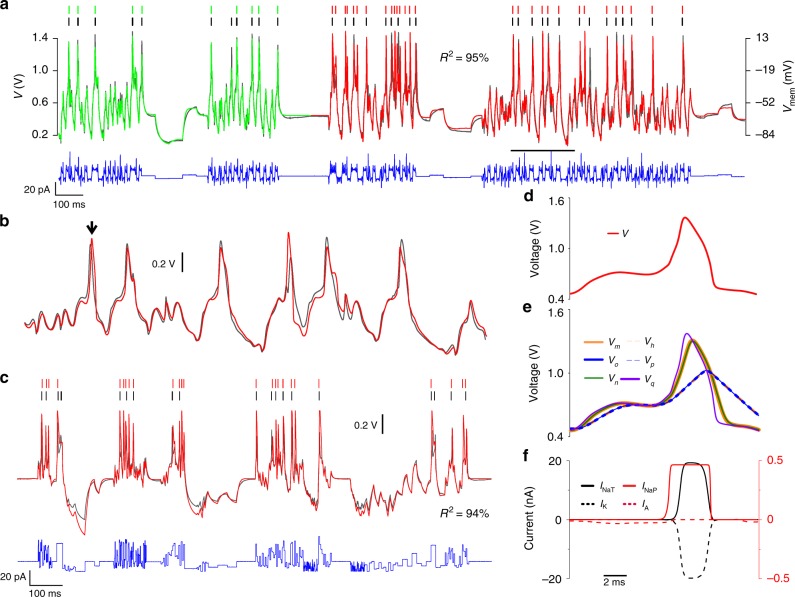
Assimilation and prediction of a respiratory neuron. **a** Intracellular recording of a respiratory neuron acquired from a slice of the Bötzinger region of the rat brain stem (black line). The neuron was stimulated with a current waveform alternating hyperchaotic oscillations and current steps (blue line). The RN SSN model was used to assimilate the experimental membrane voltage over a 920-ms-long window (green trace) to estimate the optimum parameters. **a**–**c** The completed RN SSN model predicts the membrane voltage (red traces) in quantitative agreement with observations (black traces) for a very wide range of current waveforms. Detail of: **d** an action potential, **e** gate variables and **f** ion current dynamics.

In Fig. 5a, we assimilated and predicted the membrane voltage of a respiratory neuron with the RN SSN model incorporating NaP, NaT, K, A and leak channels^55,56^. The parameters extracted from the best fit (green line) to the experimental membrane voltage (black line) are listed in Table 3 (“RN $\to$ SSN”). The RN SSN model completed with these parameters predicts the oscillations of the RN neuron to a high degree of accuracy from the end of the assimilation window (Fig. 5a). The spike coincidence factor was 71% and ${R}^{2}=95 \%$. A detailed comparison of data and prediction over the interval indicated by the horizontal bar is given in Fig. 5b. In Fig. 5c, spikes have a coincidence factor of 56% whilst there is a ${R}^{2}=94 \%$ match between the predicted and observed times series voltages. The greatest source of error arose from oscillations induced by hyperpolarizing currents—which are larger in Fig. 5c than those in the assimilation window (Fig. 5a). The accuracy of predictions to 60 different current stimuli demonstrated the successful transfer of information from the respiratory neuron to the RN SSN model. Focussing on a single action potential (Fig. 5d), we plotted the dynamics of gate variables (Fig. 5e) and ionic currents (Fig. 5f) predicted by the RN SSN model. The NaP current (${V}_{q}$) initiates depolarization with a small current that rapidly saturates at 0.5 nA. The largest contribution to the depolarizing current arises from the NaT channel. The maximum NaT current is 40 times greater than the maximum NaP current. As in the HH and CA1 examples above, the delayed K current repolarises the membrane with a residual contribution from the A current. We found that the RN SSN model accurately represented the dynamic range of the respiratory neuron and the activation sequence of its ion channels.

## Discussion

The present methodology paves the way towards making synthetic neurons to repair biocircuits of the central nervous system when their regulation of vital functions is lost to disease. For example, the respiratory neurons which we have modelled in Fig. 5 couple the respiratory and cardiac rhythms and are responsible for respiratory sinus arrythmia. Loss of this coupling through age or disease is a prognosis for sleep apnoea and heart failure^17,57^. Therefore, a device that adapts to biofeedback in the same way as respiratory neurons may offer a much needed therapy for heart failure. Our accurate description of the neurobiology within a model derived from silicon physics answers this need. Our approach combines several breakthroughs, which open new horizons to neuromorphic engineering from programming analogue computers to soft bioimplants. First, data assimilation estimates all parameters in an automated manner, which eliminates subjective criteria. Earlier methods fitted subsets of data defined by multi-objective functions^26–28^, such as the timings of action potentials^41^, the rate of fire of neurons^20^, or the sequential sampling of individual ion channels^42^. Data assimilation disentangles all model parameters from a single observation of the membrane voltage over one finite time window. In contrast, the trial-and-error method of Grassia et al.^42^ adjusts the parameters of each ion channel separately as these are individually addressed by voltage clamp experiments. This approach requires several pharmacological manipulations to probe individual ion channels and only infers a subset of model parameters. Our assimilation of large datasets presents the advantage of averaging noise and stochastic fluctuations of the membrane voltage over wide time windows. This minimizes uncertainty on extracted parameters and the wider the assimilation windows, the lower this uncertainty is. It follows that the membrane voltage predicted by such models is sufficiently accurate to reveal the occurrence of a stochastic excursion in the experimental membrane voltage through deviations from model predictions. The second breakthrough towards making quantitative predictions is our derivation of a physical model of the hardware and the demonstration of its ability to successfully assimilate biological neurons. The shortcoming of earlier approaches^39,42^ was their use of the HH model as a proxy of the hardware dynamics in the hope that parameters estimated with the HH model would give correct predictions when inserted in the hardware. For predictions to be successful, the same system of equations must be used when both assimilating data and forward integrating completed models. In this way, it is possible to also predict the time dependence of gate variables (Figs. 4 and 5). The third benefit of our approach is the versatility of the SSN model, which allows the inclusion of different types of ion channels, different activation slopes and different gate kinetics to describe complex mammalian neurons. Neuromorphic engineering often uses simplified neuron models such as the integrate-and-fire neuron^20,41^, which are inadequate for modelling actual neurons. Our approach therefore provides a robust method for faithfully transferring neuronal dynamics from a biological cell to the SSN model and from the SSN model to a chip. The respiratory neuron plays an important role for simulating the respiratory central pattern generator in bioimplants that aim to restore heart rate variability^58,59^. The SSN respiratory neuron had an average power consumption of 139 nW at a firing rate of 240 Hz and dissipated 579 pJ per spike (Supplementary Note 1, Supplementary Fig. 2). Although it was not optimized for low power consumption, our analogue neuron had a power consumption $1{0}^{9}$ times smaller than the equivalent digital implementation, which makes our approach highly suited for bioimplants.

Although our SSN model was primarily developed for sub-threshold low power silicon circuits, the sub-threshold model would also be applicable to organic transistor circuits, which rely on percolation transport and are extremely attractive for flexible implantable biocircuits. The SSN model may easily be modified to describe above-threshold circuits, for example, to implement devices with discrete electronic components. It would suffice to replace Eq. S2 with the saturation characteristics of field effect transistors in the derivation of the SSN model.

We now turn to the formal differences between Eqs. (10) and (11) and the effect of these differences on the stability of the variational optimization scheme. First, the sodium (NaT) current is restricted to positive values (${I}_{m}-{I}_{h}\,> \, 0$) by the current mirror in Fig. 1a. This current mirror introduces in Eq. (10) a Heaviside function $\theta ({I}_{m}-{I}_{h})$, which is not differentiable. For the computational model to fulfil the condition of double differentiability on ${F}_{d}[{\boldsymbol{x}}(t),{\boldsymbol{p}}]$ functions, we had to approximate the Heaviside function with a sigmoidal function. It is worth noting that alternative neuromorphic designs can make extensive use of current mirrors, hence producing models less compatible with data assimilation. For example, Rasche and Douglas^43^ substitute differential pairs with transconductance amplifiers to synthesize (in)activation curves. Current mirrors need to be added in output of these amplifiers to limit the bipolar swings of currents ${I}_{m}$ and ${I}_{h}$ to positive values, and this introduces two extra Heaviside function per ion channel (Supplementary Note 2). This makes the Rasche–Douglas model less stable than the SSN model within the optimization scheme. The extra current mirrors also truncate the (in)activation curves below the (in)activation threshold thus giving less realistic ionic current dependence on membrane voltage. Our SSN model is therefore superior since it avoids both pitfalls. A second difference between SSN and HH models is in the formal difference between the rate equations of gate variables. The gate equations of the SSN model include two state variables $V$ and ${V}_{\gamma }$ in argument of the $\tanh ()$. As we have seen in Fig. 3f, ${V}_{\gamma }$ closely follows $V$ so that one may approximate $\tanh \beta (V-{V}_{\gamma })\approx \beta (V-{V}_{\gamma })$ in Eq. (10). This closeness increases the likelihood of positive Lyapunov exponents occurring during assimilation. One way to stabilize the gate equation is to introduce a regularization term of the form $u(t)[{V}_{\gamma }-V]$ on the right-hand side of the gate equations to prevent the occurrence of positive Lyapunov exponents during optimization.

We calculated the Pearson’s correlation matrix of multiple parameter sets estimated from different assimilation windows and by setting different initial conditions to the parameter search. The smaller values of the Pearson’s correlation indicate parameters that are well constrained, while the larger values indicate “sloppy” parameters. We find that voltage thresholds and kinetic parameters are well constrained. Parameters exhibiting the greatest correlations were the maximum ionic currents ${I}_{gm}$, ${I}_{gh}$ and ${I}_{gn}$. One of these parameters was clearly a free parameter^35,37^, which could be set by specifying a narrower search interval.

As well as constraining parameters relating to transient currents, our method successfully determines the parameters of slow ionic currents, which are responsible for frequency adaptation (Fig. 4f). We have verified that the completed CA1 model replicates the adaptation and latency of the actual CA1 neuron to current steps (Supplementary Note 3 and Supplementary Fig. 3). When the slow M and NaP currents are removed from the model, the neuron fires tonically with no adaptation (Fig. 3c). Under constant current stimulation, fluctuations in the sub-threshold membrane potential are known to randomly shift the timings of biological action potentials (Supplementary Fig. 3). As a result, the response of a biological neuron to current steps lacks reproducibility. This is why the predictions of the model are best validated against the complex current protocols of Figs. 2–5.

Our patch-clamp experiments have purposely injected a current in the soma. Action potentials initiated in the soma back-propagate from the soma to distal dendrites and eventually vanish at branching points in the dendritic tree^60^. This experimental design allows the neuron to be modelled as a single compartment consisting of the excitable soma feeding into dendrites as passive transmission lines. This approach dissociates the excitable response of the dendrites from that of the soma since the former is activated by synaptic afferents on dendrites. From a computational point of view, a single compartment model is useful to keep the number of adjustable parameters to a minimum necessary to predict the membrane voltage. For this reason, the single compartment model is most effective at demonstrating the optimization method. Our use of a single compartment model is further justified by evidence that calcium channels, which are mostly concentrated in dendrites, play no role in the depolarization of the soma (Supplementary Fig. 4 and Note 4).

In vivo, however, dendrites are the predominant receiving sites for synaptic signals. Synaptic afferents may activate calcium channels in the dendrites and elicit dendritic spikes that forward propagate to the soma. A second SSN compartment would therefore have to be added to describe the active properties of dendrites^61^. The circuitry of calcium AHP channels exists (Supplementary Fig. 5) and may easily be combined^43^ with a dendritic leak channel to form a second SSN compartment (Supplementary Note 4). The size of the model to optimize would increase as a result, thus setting tighter conditions on the observability and identifiability of parameters. We have successfully assimilated a model incorporating the AHP current (Supplementary Fig. 4), showing that multi-compartment models may be similarly optimized to predict the dynamics of neurons stimulated through dendrites.

In summary, our methodology allows configuring a silicon biocircuit with an optimum parameter set that transfers the complete dynamics of a biological neuron onto a chip. This approach provides a systematic way to programme an analogue computer. It is most relevant to bioelectronic medicine where low power bioimplants are needed that adapt to physiological feedback in real time and therapies for chronic disease that rely on the repair of diseased circuits of the central nervous system.

## Methods

### Constrained nonlinear optimization

The state of a neuron at a given time is specified by its membrane voltage and the state of its ionic gates. We represent this state with a $D$-dimensional vector $x(t)=\left[x_{1}(t),\ldots ,{x}_{D}(t)\right]$, which evolves in time according to the equations of motion:

1 $$\frac{{\mathrm{d}}{x}_{d}(t)}{\mathrm{d}t}={F}_{d}[{\boldsymbol{x}}(t),{\boldsymbol{p}}],\ \ \ \ \ d=1,\ldots ,D,$$

where ${\boldsymbol{p}}=\left[{p}_{1},\ldots ,{p}_{K}\right]$ are the system parameters and the ${F}_{d}$ are given by the conductance model. The parameters hold the voltage thresholds, gate recovery times and maximum conductances of individual ion channels. Constrained nonlinear optimization is used to find the optimum set of parameters, which synchronizes state variable, ${x}_{1}(t)$, to the membrane voltage $V({t}_{i})$ observed at discrete times ${t}_{i}=iT/N$, $i=0, \ldots, N$ over an assimilation window of duration $T$. We measure the mismatch between the observation $V(t)$ and state variable ${x}_{1}(t)$ using a least-squares metric given by the following cost function:

2 $$C({\boldsymbol{x}}({t}_{0}),{\boldsymbol{p}})=\frac{1}{2N}{\mathop {\sum}\limits_{i=0}^{N}}{\left[V({t}_{i})-{x}_{1}({t}_{i})\right]}^{2}+u{({t}_{i})}^{2},$$

where $u$ is a positive control variable that nudges convergence towards the global minimum of $C({\boldsymbol{x}}({t}_{0}),{\boldsymbol{p}})$ by eliminating the occurrence of positive conditional Lyapunov exponents^62^. A regularization term $u(t)\left[{x}_{1}(t)-V(t)\right]$ is added to Eq. (1) ($d=1$) to smooth out the irregularities in ${F}_{1}\left[{\boldsymbol{x}}(t),{\boldsymbol{p}}\right]$ at large values of $u$. Within the optimization process, $u$ is treated as an additional state variable, which vanishes as the parameter search approaches the global minimum of the cost function. When the ${F}_{1},\ldots,{F}_{D}$ equations of the model are known—for example, when the membrane voltage data are synthesized by a known model—$u$ vanishes at every ${t}_{i}$ over the assimilation window and the minimization problem has a single valued solution^36^. The ${F}_{d}$s of biological neurons are, however, generally unknown. The model error makes the assimilation problem *ill-defined*. Heuristically, solutions fall into two categories. Either the parameter search arrives near the global minimum of the cost function and $u(t)$ vanishes everywhere across the assimilation window except at a few times ${t}_{i}$, or the parameter search arrives at a local minimum where the cost function is orders of magnitudes greater and $u(t)$ remains finite. In the former case, completed models incorporating the extracted parameters retain great predictive accuracy^35,37^. In the latter case, predictions invariably fail.

The problem was optimized by constructing the Karush–Kuhn–Tucker (KKT) Lagrangian^63^ incorporating the cost function (Eq. (2)) with equality and inequality constraints. The equality constraints were given by linearizing the $D$ equations of the conductance model according to Boole’s interpolation rule over four consecutive time steps of the assimilation window:

3 $${x}_{d}({t}_{i+4})={x}_{d}({t}_{i})+2h	\left[\frac{7}{45}{F}_{d}\left({\boldsymbol{x}}({t}_{i}),{\boldsymbol{p}}\right)+\frac{32}{45}{F}_{d}\left({\boldsymbol{x}}({t}_{i+1}),{\boldsymbol{p}}\right)+\frac{12}{45}{F}_{d}\left({\boldsymbol{x}}({t}_{i+2},{\boldsymbol{p}})\right)\right.\\ 	 \left.+\frac{32}{45}{F}_{d}\left({\boldsymbol{x}}({t}_{i+3}),{\boldsymbol{p}}\right)+\frac{7}{45}{F}_{d}\left({\boldsymbol{x}}({t}_{i+4}),{\boldsymbol{p}}\right)\right],$$

where $h=T/N$ (0.02 ms) is the step size. Discretization of the model equations gave $D\,\times N/4$ constraints. To prevent convergence towards physically implausible non-smooth solutions, the model equations were supplemented by $D\,\times N/4$ Hermite polynomial interpolation constraints^64^ and $N/4$ polynomial interpolation constraints for the control term, giving a total of $(2D+1)N/4$ equality constraints. Interpolation using Boole’s rule had the merit of halving the number of constraints relative to, for example, Simpson’s rule while increasing the accuracy of parameter estimation. There were $2K$ inequality constraints arising from bracketing the search interval of each parameter between a lower bound and an upper bound. The Hessian and Jacobian matrices of the cost function and constraints were calculated using symbolic differentiation (SymPy^65^). The KKT system was solved iteratively using Newton’s method until convergence was achieved^66^. Convergence was tested for the uniqueness of parameter estimates by comparing parameters extracted from different assimilation windows. Data assimilation was run on a 16-core (3.20 GHz) Linux workstation with 62.8 GB of random access memory. The completed models were forward integrated in Python 3.6 using the *odeint()* routine, which is well suited for integrating stiff systems of nonlinear equations. We built a suite of C-programs to validate forward integration against a fifth-order adaptive step-size Runge–Kutta method, to perform statistical analysis on estimated parameters, and to perform principal component analysis on the covariance matrix of extracted parameters to evaluate parameter sloppiness^37^. Links to the open source MA97 and IPOPT solvers are given in the Additional Information section.

### Biomimetic solid-state ion channel

We have designed the solid-state ion channel shown in Fig. 1a to compute the dynamics of a generic voltage-gated ion current, ${I}_{\alpha }$. The ion channel has an activation gate ($m$) and an inactivation gate ($h$). The maximum ionic conductances and recovery time constants are, respectively, set by source currents ${I}_{g\gamma }$ and ${I}_{\tau \gamma }$ and ${I}_{T\gamma }$. The gate voltage thresholds are ${V}_{t\gamma }$ where $\gamma \in \left\{m,h\right\}$. The equations of motion of individual ionic gates are derived from analysis of the circuit in Fig. 1a, whereas the equation of motion of the membrane voltage is obtained from the electrical equivalent circuit of the neuron membrane in Fig. 1b. The ion channel is then configured to mimic individual types of biological ion channels (Table 2) using estimated parameters as we shall see below. As many such channels as specified by the assimilation filter are then added to the neuron membrane circuit.

Whole-cell current-clamp recordings of biological neurons show that the gate recovery time depends on the membrane voltage^45^. This dependence, $\tau (V)={t}_{0}+\epsilon \left[1-{\tanh }^{2}\frac{V \,- \,{V}_{t}}{\delta {V}_{\tau }}\right]$^36^, is a bell-shaped curve of width $\delta {V}_{\tau }$ centred on the (in)activation threshold ${V}_{t}$. The base latency time is ${t}_{0}$ and peak latency time ${t}_{0}+\epsilon$. The voltage dependence of ${\tau }_{\gamma }(V)$, $\gamma \in \left\{m,h\right\}$, is modelled with current ${I}_{\Sigma }\gamma$ in Fig. 1a. The bell-shaped dependence is obtained by connecting in series n-type and p-type differential pair circuits (Fig. 1c). The n-type differential pair outputs a sigmoidal current $I^{\prime} ={I}_{\mathrm{max}}/2\left[1+\tanh \beta (V-{V}_{t\gamma })\right]$ (Supplementary Note 5, Supplementary Fig. 6), which supplies the source current of the p-type differential pair $I=I^{\prime} /2\left[1+\tanh \beta ({V}_{t}-V)\right]$. The product of activating and inactivating characteristics thus produces the bell-shaped voltage dependence of ${I}_{oT\gamma }$. By adding a constant current ${I}_{\tau \gamma }$ to ${I}_{oT\gamma }$, one obtains:

4 $${I}_{\Sigma \gamma }={I}_{\tau \gamma }+\frac{{I}_{T\gamma }}{4}\left[1-{\tanh }^{2}\beta (V-{V}_{t\gamma })\right],$$

which has the same voltage dependence as ${\tau }_{\gamma }(V)$, $\gamma \in \left\{m,h\right\}$. This result assumes sub-threshold transistors^44^ for which $\beta =\kappa /(2{U}_{T})\approx 14$ V^−1^, ${U}_{T}\,\approx\,25\,{\mathrm{mV}}$ is the thermal voltage, $\kappa ={C}_{\mathrm{Ox}}/({C}_{\mathrm{Ox}}+{C}_{\mathrm{D}})\approx 0.7$ and, ${C}_{\mathrm{Ox}}$ (${C}_{\mathrm{D}}$) is the capacitance of the oxide (depletion) layer.

${I}_{\Sigma \gamma }$ is injected in one input of an analogue current multiplier, which requires some transistors to be biased in the above-threshold region (Fig. 1e)^67^. The other input receives the displacement current through capacitor ${C}_{\gamma }$: ${I}_{C\gamma }={C}_{\gamma }{\mathrm{d}}{V}_{\gamma }/{\mathrm{d}}t$, (Fig. 1a). The output of the current multiplier is ${I}_{C\gamma }={I}_{\times\! \gamma }\times {I}_{\tau \gamma }/{I}_{\Sigma \gamma }$. A current mirror drains ${I}_{\times\! \gamma }$ to ground and equates it to the current output by the transconductance amplifier: ${I}_{\tau \gamma }\tanh \beta (V-{V}_{\gamma })$ (Fig. 1f, Supplementary Note 5, Supplementary Figs. 7, 8). This analogue circuit determines the rate of change of gate variable, ${V}_{\gamma }$. Substituting Eq. (4) in the equality ${I}_{C\gamma }={I}_{\tau \gamma }\times {I}_{\tau \gamma }\tanh \beta (V-{V}_{\gamma })/{I}_{\Sigma \gamma }$, one obtains the following equation of motion for the gate variable:

5 $${C}_{\gamma }\frac{{\mathrm{d}}{V}_{\gamma }}{{\mathrm{d}}t}=\frac{{I}_{\tau \gamma }\tanh \beta (V-{V}_{\gamma })}{1+\frac{{I}_{T\gamma }}{4{I}_{\tau \gamma }}\left[1-{\tanh }^{2}\beta (V-{V}_{t\gamma })\right]}.$$

${V}_{\gamma }$ is the membrane voltage delayed by a recovery time identical to ${\tau }_{\gamma }(V)$. The delayed voltage is then input into a sigmoidal circuit (Fig. 1g), which generates gate current ${I}_{\gamma }$ (Supplementary Note 5):

6 $${I}_{\gamma }=\frac{{I}_{g\gamma }}{2}\left[1+\tanh \beta ({V}_{\gamma }-{V}_{t\gamma })\right].$$

Equations (5) and (6) are exact solutions derived from the circuit analysis. It is useful to compare these equations with the equations of gate variables in conductance models:

7 $$\frac{{\mathrm{d}}\gamma }{{\mathrm{d}}t}=\frac{{\gamma }_{\infty }(V)-\gamma }{{\tau }_{\gamma }(V)},$$

where ${\gamma }_{\infty }(V) \, = \, 0.5[1+\tanh ((V-{V}_{t\gamma })/\delta {V}_{\gamma })]$ and ${\tau }_{\gamma }(V)\, = \, {t}_{0,\gamma }+ {\epsilon }_{\gamma } [1-{\tanh }^{2}((V-{V}_{t\gamma })/\delta {V}_{\tau \gamma })]$. In the limit $V\approx {V}_{\gamma }$, which corresponds to the domain of operation of the circuit (see Discussion section), Eq. (5) becomes identical to Eq. (7) once the change of variable ${\gamma }_{\infty }(V)\equiv V/{U}_{T}$ and $\gamma \equiv {V}_{\gamma }/{U}_{T}$ is made. The gate recovery times of the conductance model are then given by ${t}_{0,\gamma }=2{C}_{\gamma }{U}_{T}/(\kappa {I}_{\tau \gamma })$ and ${\epsilon }_{\gamma }={C}_{\gamma }{U}_{T}{I}_{T\gamma }/(2\kappa {I}_{\tau \gamma }^{2})$.

In actual neurons, the width of the transition region from the open to the closed state of the gate, $\delta {V}_{\gamma }$, and the width of the bell-shaped recovery time, $\delta {V}_{\tau \gamma }$, vary from one type of ion channel to another. The corresponding parameter in the SSN equations Eqs. (5) and (6) is ${\beta }^{-1}\approx 71.4$ mV. For the circuits of Fig. 1, these values are fixed by processing technology. However, by changing circuit design it is possible to modify them. This is essential to adequately emulate biological properties. Another factor that effectively increases the slope of (in)activation curves is the exponent of gate variables in the equation of ionic currents: ${I}_{\alpha }={\bar{g}}_{\alpha }{m}^{a}{h}^{b}({E}_{\alpha }-V)$, where ${\bar{g}}_{\alpha }$ is the maximum conductance and ${E}_{\alpha }$ the ion reversal potential (Table 2). To first order, the exponents $a$ and $b$ increase the slope of transition regions from $1/\delta {V}_{m}\to a/\delta {V}_{m}$ and $1/\delta {V}_{h}\to b/\delta {V}_{h}$ while shifting the *effective* voltage thresholds ${V}_{tm}$ and ${V}_{th}$ higher. It is therefore essential to incorporate additional flexibility in the design of Fig. 1a to allow for variable activation slopes $\beta \to {\beta }_{m}$ and inactivation slopes $\beta \to {\beta }_{h}$. It is equally desirable to make the width of the bell-shaped kinetics adjustable through $\beta \to {\beta }_{\tau m}$ and $\beta \to {\beta }_{\tau h}$. In the next section, we describe the circuit modifications made to Fig. 1a to introduce adjustable activation slopes ${\beta }_{\gamma }\equiv a/\delta {V}_{\gamma }$ and kinetics ${\beta }_{\tau \gamma }\equiv 1/\delta {V}_{\tau \gamma }$ to describe the different types of ionic currents in Table 2.

### Analogue interpolation of activation curves and gate kinetics

**Fig. 6:**
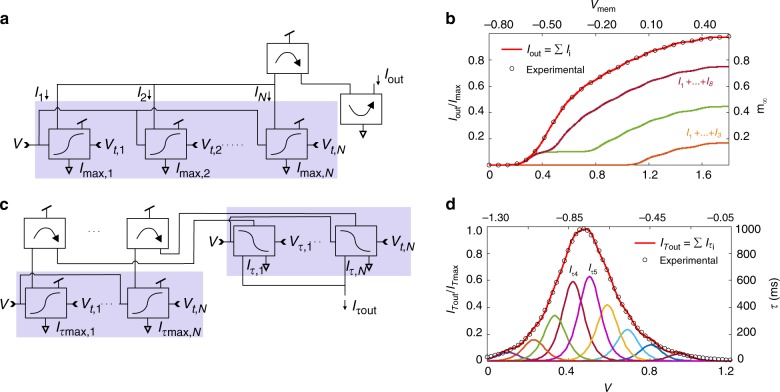
Analogue interpolation of the gate activation curves and gate kinetics. **a** Sigmoidal currents ${I}_{1}+\cdots +{I}_{N}$ are summed to interpolate the activation curve of an ionic gate. The adjustment parameters are the voltage thresholds ${V}_{t1},\ldots ,{V}_{tN}$ and source currents ${I}_{\mathrm{max},1},\ldots ,{I}_{\mathrm{max},N}$. **b** Activation curve of the A channel of a thalamic relay neuron (circle symbols)^45^ interpolated by nine sigmoids whose sum gives the output current ${I}_{\mathrm{out}}$ (full red line). The output current normalized by ${I}_{\mathrm{max}}={I}_{\mathrm{max},1}+\cdots +{I}_{\mathrm{max},9}$ gives the biological activation curve, ${m}_{\infty }(V)$. **c** Circuit interpolating the activation/inactivation kinetics by summing $N$ bell-shaped curves centred at ${V}_{t1},\ldots ,{V}_{tN}$ with amplitudes ${I}_{\mathrm{max},1},\ldots ,{I}_{{\mathrm{max}},N}$. **d** Activation kinetics of the HCN current, $\tau (V)$,^45^ (circle symbols) interpolated by summing nine bell-shaped curves in the output current ${I}_{\tau {\mathrm{out}}}$ (full red line). ${I}_{\tau {\mathrm{max}}}={I}_{\tau {\mathrm{max}},1}+\cdots +{I}_{\tau {\mathrm{max}},9}$.

To emulate a variable slope ${\beta }_{\gamma }$ and more generally activation curves of arbitrary shape, we designed a new circuit composed of multiple differential pairs and superposed their currents (Fig. 6a). The differential pairs are biased at different voltage thresholds ${V}_{t\gamma ,i}$, and saturation currents ${I}_{{\mathrm{max}},i}$, $i=1, \ldots, n$^39^. The sum of their currents interpolates the activation (inactivation) according to:

8 $${I}_{\gamma }={\mathop {\sum}\limits_{i=1}^{n}}\frac{{I}_{g\gamma ,i}}{2}\left[1+\tanh \beta ({V}_{\gamma }-{V}_{t\gamma ,i})\right].$$

Figure 6b shows an example of interpolation of the activation curve of the A channel of a thalamic relay neuron as measured by McCormick and Huguenard^45^. We used $n=9$ differential pairs to interpolate this activation curve (open dots) to excellent accuracy (solid red line). The summations of the first 3, 6 and 8 currents ${I}_{i}$ are also shown (dashed lines). Our SSN circuit thus replaces the static activation circuit of Fig. 1a with the interpolation circuit of Fig. 6a. Correspondingly, we replaced the fixed activation slope $\beta$ with ${\beta }_{\gamma }$ in the SSN model (Supplementary Fig. 9). The source currents ${I}_{g\gamma ,i}$ of the interpolating circuit satisfy the sum rule ${I}_{g\gamma }={\sum }_{i=1}^{n}{I}_{g\gamma ,i}$.

Similarly, the width of the voltage-dependent kinetics ${\beta }_{\tau \gamma }$ is made to vary by superposing the currents of $n$ bell-shaped generating circuits (Fig. 6c, Supplementary Fig. 10), which peak at thresholds ${V}_{t\gamma ,i}$ spanning a range of voltages and source currents ${I}_{T\gamma ,i}$. A bell-shaped current of given width at half-maximum is synthesized as:

9 $${I}_{oT\gamma }={\mathop{\sum}\limits_{i=1}^{n}}{I}_{T\gamma ,i}\left[1-{{\tanh}}^{2}\beta (V-{V}_{t\gamma ,i})\right].$$

Figure 6d shows the voltage-dependent inactivation kinetics of the HCN current (open dots) measured by Huguenard and McCormick^45^ in a thalamic relay neuron. This dependence is fitted (full red line) by summing $n=9$ bell-shaped current curves (dashed lines). The bell-shaped generating circuit in Fig. 1a is to be replaced with Fig. 6c.

### SSN model

The equation of motion of the SSN model may now be written out by replacing $\beta$ with the interpolated ${\beta }_{\gamma }$ in Eq. (6) and $\beta$ with ${\beta }_{\tau \gamma }$ in the denominator of Eq. (5). The rate of change of the membrane voltage is obtained from Kirchhoff’s current and voltage laws applied to the electrical equivalent circuit of the neuron membrane (Fig. 1b). A SSN incorporating Na, K and leak channels ($D=4$) has the following equations:

10 $$C\frac{{\mathrm{d}}V}{{\mathrm{d}}t}	= ({I}_{m}-{I}_{h})\ \theta ({I}_{m}-{I}_{h})-{I}_{n}+{I}_{\mathrm{L}}\tanh {\beta }_{\mathrm{L}}({E}_{\mathrm{L}}-V)+\alpha {I}_{\mathrm{inj}}+{I}_{\mathrm{dark}},\\ \frac{{\mathrm{d}}{V}_{m}}{{\mathrm{d}}t}	= \frac{{\tilde{I}}_{\tau m}\tanh \beta (V-{V}_{m})}{1+\frac{{\tilde{I}}_{Tm}}{4{\tilde{I}}_{\tau m}}\left[1-{\tanh }^{2}{\beta }_{\tau m}(V-{V}_{tm})\right]}\ \ ,\ \ {I}_{m}=\frac{{I}_{gm}}{2}\left[1+\tanh {\beta }_{m}({V}_{m}-{V}_{tm})\right],\\ \frac{{\mathrm{d}}{V}_{h}}{{\mathrm{d}}t}	= \frac{{\tilde{I}}_{\tau h}\tanh \beta (V-{V}_{h})}{1+\frac{{\tilde{I}}_{Th}}{4{\tilde{I}}_{\tau h}}\left[1-{\tanh }^{2}{\beta }_{\tau h}(V-{V}_{th})\right]}\ \ ,\ \ {I}_{h}=\frac{{I}_{gh}}{2}\left[1+\tanh {\beta }_{h}({V}_{h}-{V}_{th})\right],\\ \frac{{\mathrm{d}}{V}_{n}}{{\mathrm{d}}t} 	= \frac{{\tilde{I}}_{\tau n}\tanh \beta (V-{V}_{n})}{1+\frac{{\tilde{I}}_{Tn}}{4{\tilde{I}}_{\tau n}}\left[1-{\tanh }^{2}{\beta }_{\tau n}(V-{V}_{tn})\right]}\ \ ,\ {I}_{n}=\frac{{I}_{gn}}{2}\left[1+\tanh {\beta }_{n}({V}_{n}-{V}_{tn})\right],$$

where $\theta ()$ is the Heaviside step function. Because the rate of change of gate variables in Eq. (5) depends on ${C}_{\gamma }$ and ${I}_{\tau \gamma }$, one defines the ratios ${\tilde{I}}_{\tau \gamma }={I}_{\tau \gamma }/{C}_{\gamma }$, $\gamma \in \left\{m,h,n\right\}$. For a VLSI designer, this has the advantage that capacitances may be made as small as convenient provided that the source current is decreased accordingly. A typical gate recovery time of 0.1–100 ms would be implemented in the solid-state with a source current of ${I}_{\tau \gamma }=0.26\! -\!\! 260$ pA and a capacitance of ${C}_{\gamma }=1$ pF. It follows that the NaKL SSN model has 22 independent parameters: ${I}_{g{\mathrm{L}}}$, ${I}_{g\gamma }$, ${\tilde{I}}_{\tau \gamma }$, ${\tilde{I}}_{T\gamma }$, ${\beta }_{\mathrm{L}}$, ${\beta }_{\gamma }$, ${\beta }_{\tau \gamma }$, ${E}_{\mathrm{L}}$, ${V}_{t\gamma }$ for $\gamma \in \left\{m,h,n\right\}$. We have introduced parameter ${I}_{\mathrm{dark}}$ to account for the leakage current of sub-threshold circuits in the OFF state. This current ($\approx10$ pA) is generally small compared to ionic currents. We also introduced the scaling parameter $\alpha$ to amplify the current injection protocol as the membrane voltage was re-scaled from the biological range [−100, +45 mV] to [0, ${V}_{dd}$] where ${V}_{dd}=1.8$ V. This parameter also accounts for the surface area of the neuron which receives the injection current.

The NaKL SSN model presents obvious similarities with the HH conductance model^1^:

11 $$C\frac{{\mathrm{d}}V}{{\mathrm{d}}t}	= {g}_{\mathrm{Na}}{m}^{3}h\left({E}_{\mathrm{Na}}-V\right)+{g}_{\mathrm{K}}{n}^{4}\left({E}_{\mathrm{K}}-V\right)+{g}_{\mathrm{L}}\left({E}_{\mathrm{L}}-V\right)+{J}_{\mathrm{inj}},\\ \frac{{\mathrm{d}}m}{{\mathrm{d}}t}	= \frac{{m}_{\infty }(V)-m}{{t}_{0,m}+{\epsilon }_{m}\left[1-{\tanh }^{2}\frac{V \, - \, {V}_{tm}}{\delta {V}_{\tau m}}\right]},\quad {m}_{\infty }(V)=0.5\left[1+\tanh \frac{V-{V}_{tm}}{\delta {V}_{m}}\right],\\ \frac{{\mathrm{d}}h}{{\mathrm{d}}t}	= \frac{{h}_{\infty }(V)-h}{{t}_{0,h}+{\epsilon }_{h}\left[1-{\tanh }^{2}\frac{V \, - \, {V}_{th}}{\delta {V}_{\tau h}}\right]},\quad{h}_{\infty }(V)=0.5\left[1+\tanh \frac{V-{V}_{th}}{\delta {V}_{h}}\right],\\ \frac{{\mathrm{d}}n}{{\mathrm{d}}t}	= \frac{{n}_{\infty }(V)-n}{{t}_{0,n}+{\epsilon }_{n}\left[1-{\tanh }^{2}\frac{V \, - \, {V}_{tn}}{\delta {V}_{\tau n}}\right]},\quad {n}_{\infty }(V)=0.5\left[1+\tanh \frac{V-{V}_{tn}}{\delta {V}_{n}}\right].$$

The main difference between both models is the order in which delay and threshold are applied to the membrane voltage as this order is inverted. Additional ionic currents $({I}_{m}-{I}_{h})\ \theta ({I}_{m}-{I}_{h})$ may be inserted in Eq. (10) in the same way as the ${g}_{\alpha }{m}^{a}{h}^{b}\left({E}_{\alpha }-V\right)$ in Eq. (11) together with the corresponding rate equations for gate variables. This versatility in principle allows modelling the most complex neurons. A universal model incorporating all the ion channels of Table 2 may be built without a priori knowledge about biology since absent ion channels would be assigned a null current by the assimilation filter^35,37^.

From the mathematical viewpoint of model optimization, both the SSN equations (Eq. (10)) and the HH equations (Eq. (11)) are approximations of the exact equations of a biological neuron that are unknown. The SSN and HH equations are, however, sufficiently close to the true equations of a biological neuron for excellent predictions to be made. Because of formal differences in the HH and SSN systems of equations, completed SSN and HH model of the same biological neuron will have different parameters. The advantage of the SSN model over the HH model, however, is that its equations are identical to those of the hardware. Therefore, the parameters of completed SSN models will be the only ones that can be used to directly configure the hardware and make it behave exactly like the biological neuron as we will show below.

### Electrophysiological protocols

#### Current injection protocols

Current injection protocols were designed to optimally constrain the model parameters and fulfil the requirements of Takens’ theorem^68^. These protocols include (i) current steps of different duration to probe the different recovery times of ion channels, and (ii) currents of different amplitudes so that information is extracted from the depolarized, sub-threshold and hyperpolarized states of the neuron. Each protocol was 10 s long and comprised various sequences of depolarizing and hyperpolarizing current steps mixed with hyperchaotic current oscillations generated using the $x$ variable of the following system^69^:

12 $$\frac{{\mathrm{d}}x}{{\mathrm{d}}t}	=\; x(1-y)+\zeta z,\\ \frac{{\mathrm{d}}y}{{\mathrm{d}}t} 	=\; \rho ({x}^{2}-1)y,\\ \frac{{\mathrm{d}}z}{{\mathrm{d}}t} 	=\; \gamma (1-y)v,\\ \frac{{\mathrm{d}}v}{{\mathrm{d}}t} 	=\; \eta {z}$$

for $(\zeta ,\rho ,\gamma ,\eta )=(-2,1,0.2,1)$. Chaotic or hyperchaotic injection currents play an important role in making the state of the neuron at a given time less dependent on the past steps that led to that state. The removal of the memory of past events (Markov condition) is important to decouple the optimization constraints from one another. These constraints are obtained by linearizing Eq. (1) at each time point of the assimilation window. Assimilation protocols had the spectrum of a low pass filter with a 8 kHz cut-off frequency. This frequency was greater than the fastest of the gate recovery rates in the biological neurons which we probed. In total, each neuron was subjected to a 10-min-long sequence of 60 protocols. These protocols allowed testing the predictive accuracy of completed models over a variety of stimuli.

#### Current-clamp recordings

Current-clamp recordings were performed using acute brain slices from male Han Wistar rats at P1–3 (respiratory neurons) and P16–17 (hippocampal neurons). Following decapitation, the brain was removed into ice-cold slicing solution composed of (mM): NaCl 52.5; sucrose 100; glucose 25; NaHCO_3_ 25; KCl 2.5; CaCl_2_ 1; MgSO_4_ 5; NaH_2_PO_4_ 1.25; kynurenic acid 0.1, and carbogenated using 95% O_2_/5% CO_2_. A Campden 7000 smz tissue slicer (Campden Instruments UK) was used to prepare transverse hippocampal slices at 350 μm, or rhythmically active transverse medullary slices at 400 μm, containing the pre-Bötzinger complex as well as the hypoglossal motor nucleus (XII) and rootlets. Slices were transferred to a submersion chamber containing carbogenated artificial cerebrospinal fluid (aCSF) composed of (mM): NaCl 124; glucose 30; NaHCO_3_ 25; KCl 3 (or above, as specified); CaCl_2_ 1.5; MgSO_4_ 1; NaH_2_PO_4_ 0.4, and incubated at 30 °C for 1–5 h prior to use.

Brainstem slices were transferred to the stage of an Axioskop 2 upright microscope (Carl Zeiss) and respiratory neurons were visualized using differential interference contrast optics. The chamber was perfused with carbogenated aCSF (composition as above) at 2 ml min^−1^ at 30 ± 2 °C. Patch pipettes were pulled from standard walled borosilicate glass (GC150F, Warner Instruments) to a resistance of 7–10 MΩ and filled with an intracellular solution composed of (mM): potassium gluconate 130; sodium gluconate 5, HEPES 10; CaCl_2_ 1.5; sodium phosphocreatine 4; Mg-ATP 4; Na-GTP 0.3; pH 7.3.

A custom-built LabView interface injected current protocols into neurons through a USB-6363 DAQ card and a MultiClamp 700B amplifier. Time series membrane voltage and current data were simultaneously recorded in current-clamp mode at 100 kHz in response to the clamp protocols (Supplementary Fig. 11). Inspiratory respiratory neurons were identified by anatomical location and the presence of burst firing activity in phase with activity in the XII motor nucleus or rootlet. To achieve this, extracellular [K^+^] was temporarily raised to 7–9 mM. Experiments measuring neuronal activity in response to the protocols applied in current-clamp mode were made in the presence of (μM) kynurenate 3, picrotoxin 0.05, and strychnine 0.01 to isolate the neuron from synaptic inputs from other neurons in the slice.

#### Model and VLSI data

Model data were synthesized with the HH model calibrated for a thalamic relay neuron^45,46^. Model parameters are listed in Supplementary Table 1. The time series evolution of the neuron state $x(t)=\left\{V(t),m(t),h(t),n(t)\right\}$ was computed by integrating current protocols with Eq. (11). The membrane voltage time series was assimilated with the SSN model (Eq. (10)). This approach allowed the dynamics of HH gate variables—which cannot be observed in real neurons—to be compared with the dynamics of gate variables in the SSN model.

The membrane voltage observed in biological neurons was re-scaled from [−100, +45 mV] to [0, 1.8 V] to fit the voltage range of the VLSI chip hosting the SSN microcircuits (Supplementary Fig. 12). The conversion formula from the observed membrane voltage (${V}_{\mathrm{mem}}$) to the SSN membrane voltage $V$ was $V\;(\mathrm{mV})=12.414\times {V}_{\mathrm{mem}}\;(\mathrm{mV})+1241.4$.

### Ethical statement

All experiments were performed under Schedule 1 in accordance with the United Kingdom Scientific Procedures (act of 1986).

## Supplementary information

Supplementary Information

## Data Availability

All data generated or analysed during this study are included in this published article and its supplementary information files. Additional requests for data may be made from the authors.

## Code availability

Exemplar equation and specification files may be requested from A.R.Nogaret@bath.ac.uk. The optimization code [www.coin-or-org/ipopt] implemented the MA97 linear solver [http://www.hsl.rl.ac.uk/catalogue]. Completed models were forward integrated with the odeint() routine [http://numerical.recipes or www.python.org].
