# Supplementary material for: Optimal solid state neurons
Source: Nat Commun. 2019 Dec 3;10:5309. doi: 10.1038/s41467-019-13177-3 (PMC6890780; doi:10.1038/s41467-019-13177-3)
Supplement: Supplementary file 1 — Supplementary Information [file 41467_2019_13177_MOESM1_ESM.pdf]

Optimal Solid State Neurons, Abu-Hassan et al.

Supplementary Information

Optimal Solid State Neurons

Kamal Abu-Hassan[1], Joseph D. Taylor[1], Paul G. Morris[1,2], Elisa Donati[3], Zuner A. Bortolotto[2], Giacomo Indiveri[3], Julian F. R. Paton[2,4] and Alain Nogaret[1]

[1] *Department of Physics, University of Bath, Bath, BA2 7AY, UK*

[2] *School of Physiology, Pharmacology and Neuroscience, University of Bristol, University Walk, Bristol BS8 1TD, UK*

[3] *Institute of Neuroinformatics, University of Zurich and ETH Zurich, Winterthurerstrasse 190, CH-8057, Switzerland*

[4] *Department of Physiology, Faculty of Medical and Health Sciences, University of Auckland, Auckland, New Zealand*

Table of Contents

I. Supplementary Figures 3

II. Supplementary Tables 11

III. Supplementary Notes 11

Supplementary Note 1: Power consumption of the VLSI neurons (SPECTRE calculations) 11

Supplementary Note 2: Solid state neuron variants 12

A. Voltage dependent gate recovery times 12

B. Rasche-Douglas variant implementing activation curves with transconductance amplifiers 12

Supplementary Note 3: Adaptation of the CA1 neuron model to current steps 13

Supplementary Note 4: Does the soma of CA1 neuron incorporate calcium channels? 13

Supplementary Note 5: First principle analysis of silicon neuron circuits 14

A. Equations of the differential pair 14

B. Equations of the transconductance amplifier 16

C. Equations of the leak current 16

D. Equations of the after-hyperpolarization current (AHP) 17

Supplementary References 17

I. Supplementary Figures

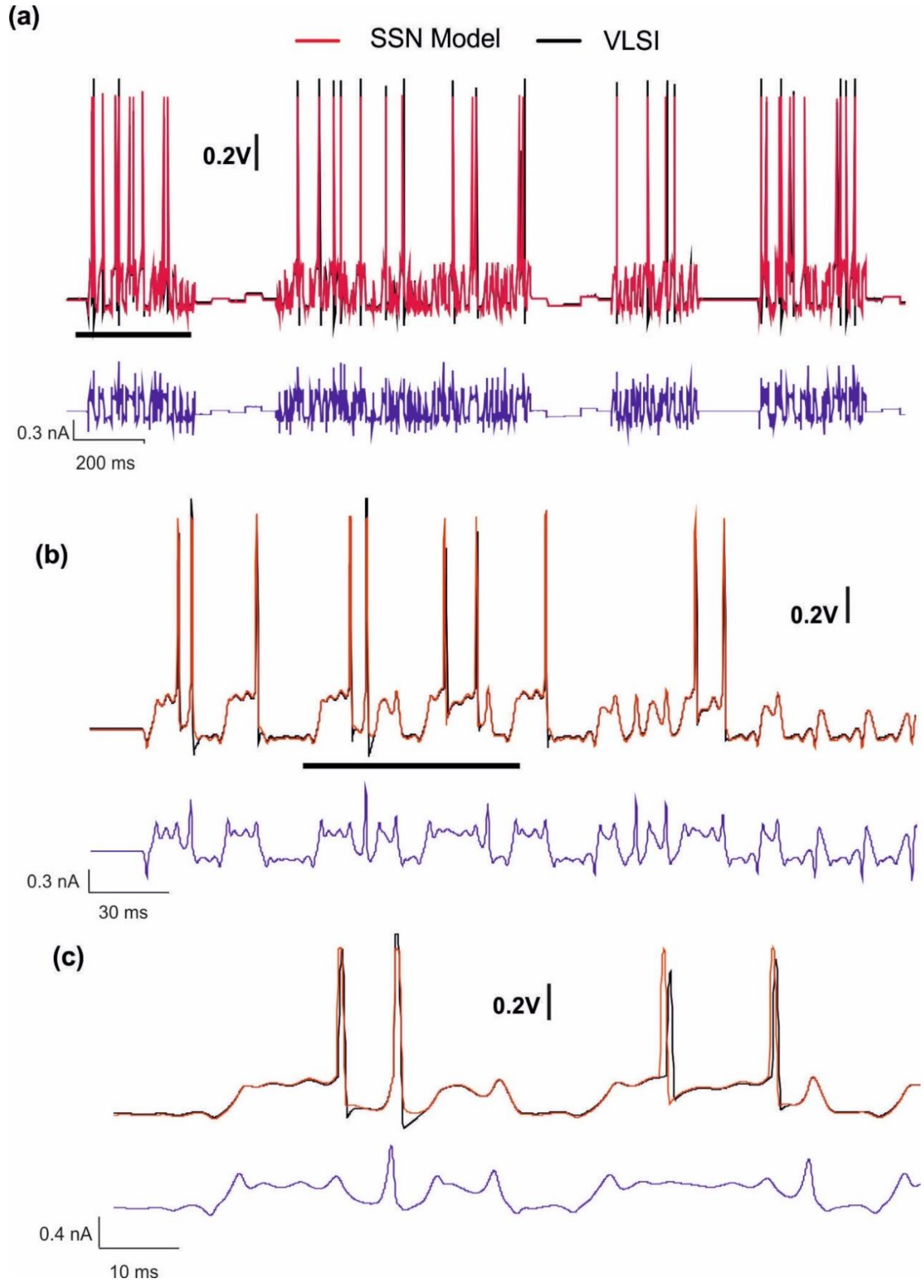

Supplementary Figure 1: Predictions of the NaKL SSN model and VLSI neuron.
(a) Voltage predicted by the SSN model (red curve) and the VLSI circuit (black curve) in response to a chaotic current protocol. Both biased with the VLSI parameter set of Table II. (b) Data corresponding to a smaller window (black bar, panel (a)), and (c) further enlarged to distinguish between the VLSI and SSN model predictions.

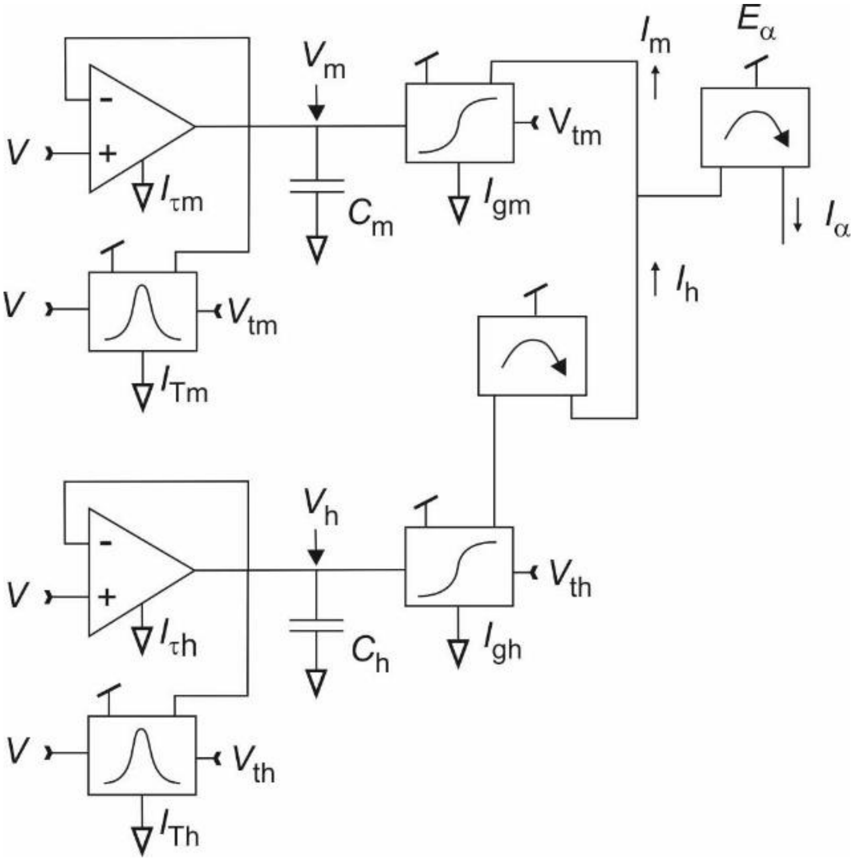

Supplementary Figure 2: Solid state ion channel implementing constant recovery time constants.
Circuit diagram of an ion channel including an activation gate (m) and an inactivation gate (h). The gate recovery time constants are given by $\tau_\gamma = \frac{2C_\gamma U_T}{\kappa I_{\tau\gamma}}$, $\gamma \equiv \{m, h\}$.

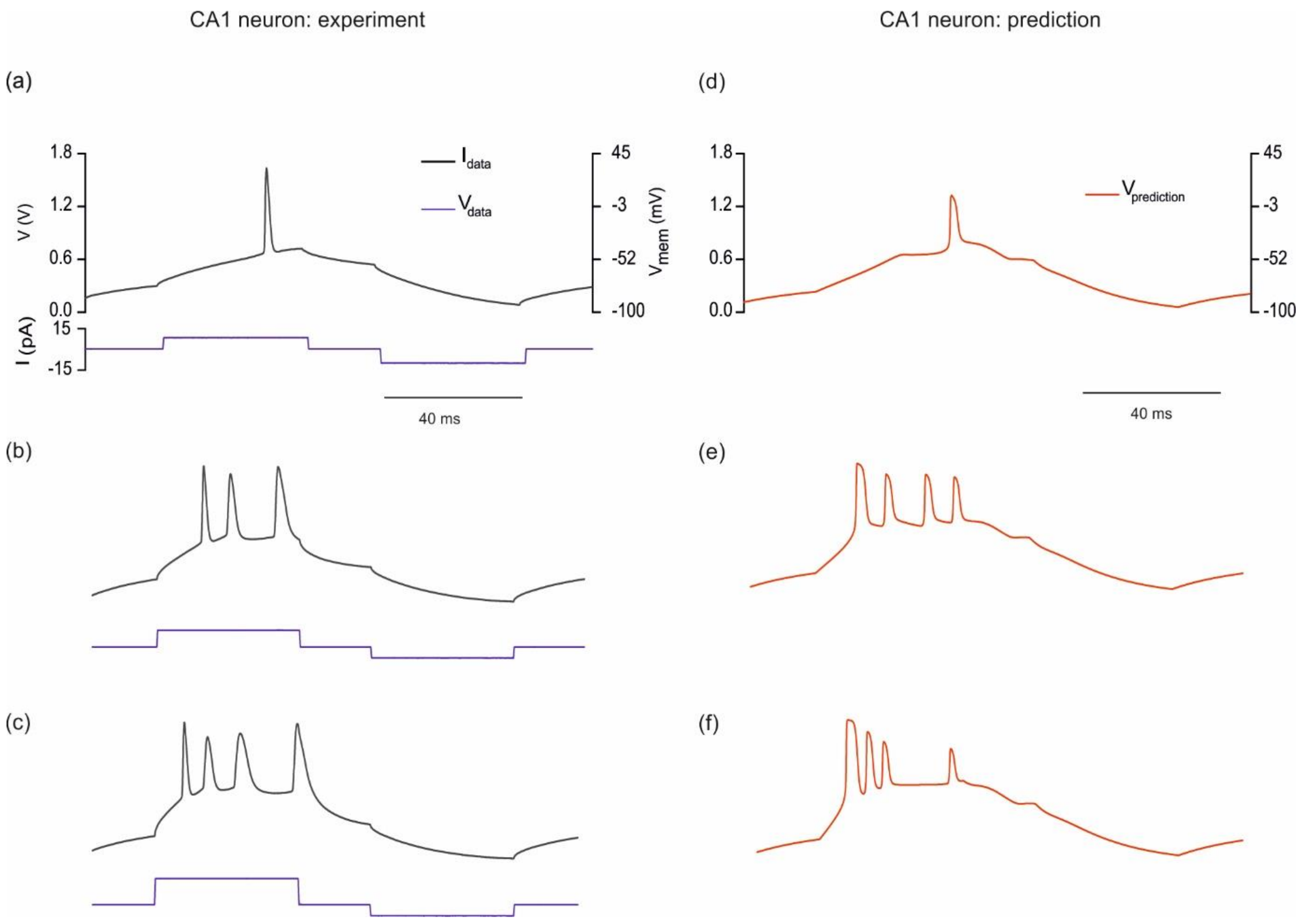

Supplementary Figure 3: Response of a CA1 neuron (left) and completed CA1 model (right) to square current steps. Each depolarisation current step was applied for 40ms after a 20ms delay. This was followed by a constant hyperpolarising step of -120pA which was applied between 80ms and 120ms. The amplitudes of the depolarising current steps in panels (a), (b) and (c) were 0.09nA, 0.19nA and 0.29nA, respectively. The completed CA1 model was forward integrated with the three current protocols to produce the voltage predictions in panels (d)-(f).

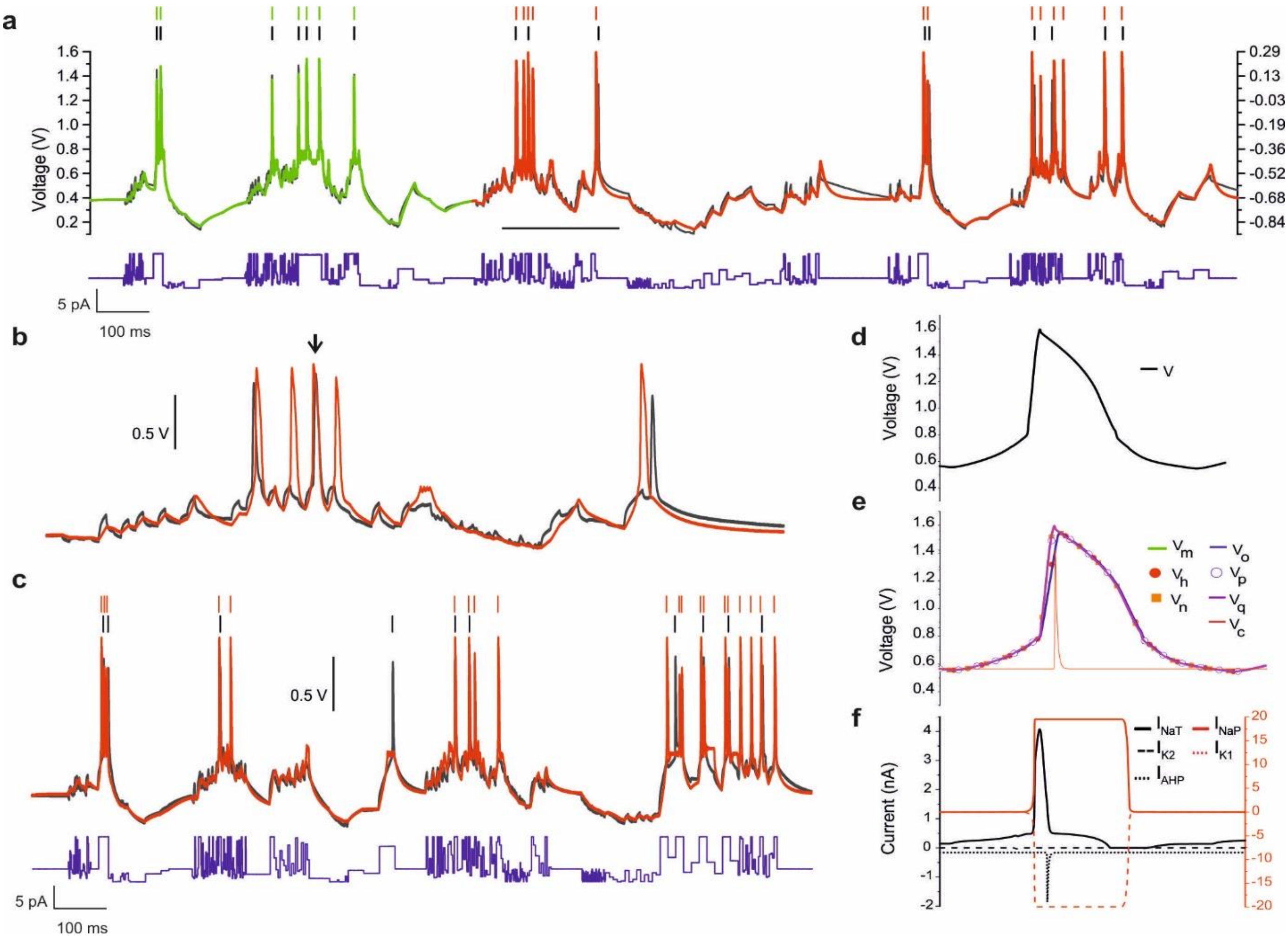

Supplementary Figure 4: Assimilation and prediction of the membrane voltage oscillations of a CA1 cell.
The CA1 model incorporates the calcium AHP channel in place of the muscarinic channel.

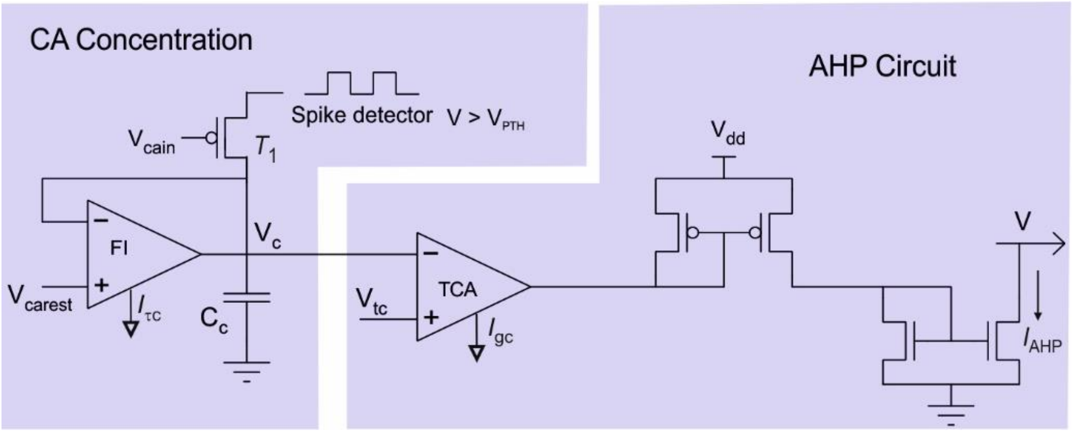

Supplementary Figure 5: Circuit of the calcium-dependent potassium current (AHP) - after Rasche and Douglas (2000)[1]**.**

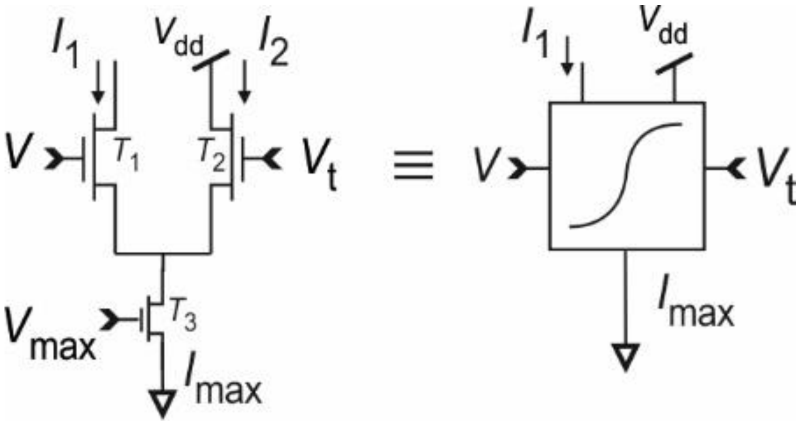

Supplementary Figure 6: Schematic of the differential transistor pair generating a sigmoidal current.
The gate voltage of transistor T_1 modulates current I_1 as it crosses the voltage threshold V_t set on transistor T_2.

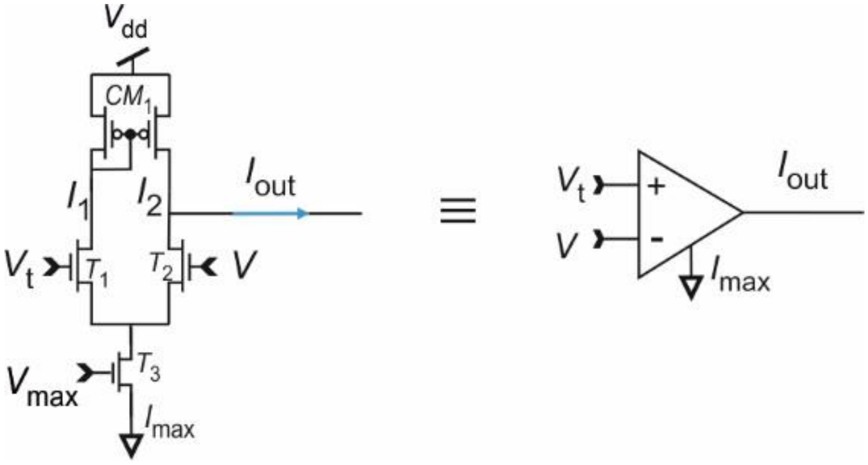

Supplementary Figure 7: Schematics of a transconductance amplifier (TCA).
The TCA combines the differential pair with a current mirror (CM1) to output the current difference $(I_1 - I_2)$.

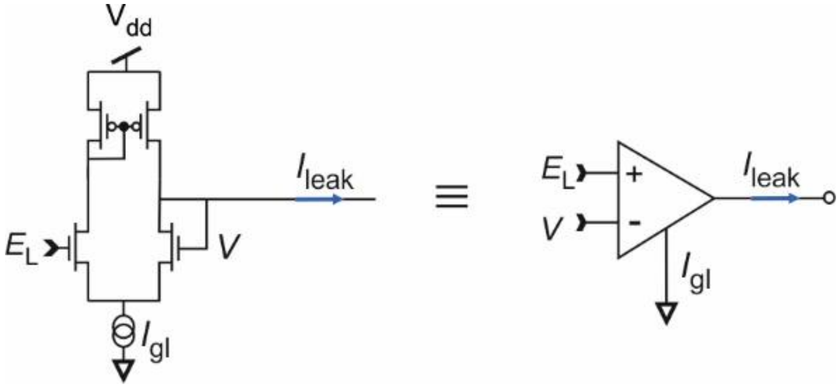

Supplementary Figure 8: Circuit diagram for the leak current.
This ion channel gives a passive conductance and a residual leak current that changes sign at the reversal potential (E_L). *V* is the membrane voltage. I_{gL} is the maximum value of the leak current.

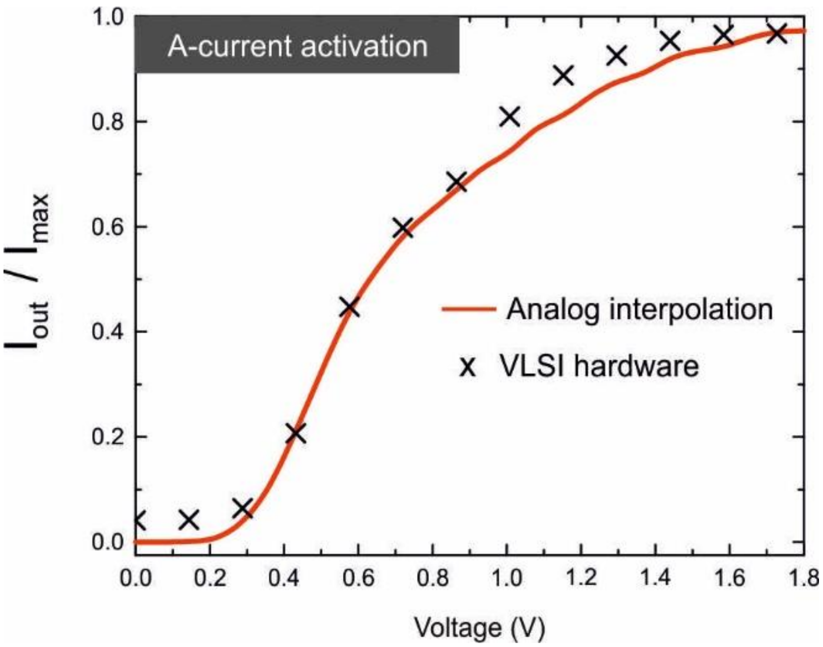

Supplementary Figure 9: Activation curve of the A ion channel of a thalamic relay neuron
The red line shows the activation curve predicted by Eq.8. The cross symbols show the same activation curve synthesized by the 9 stage circuit of Fig.2(a) as simulated in Spectre®.

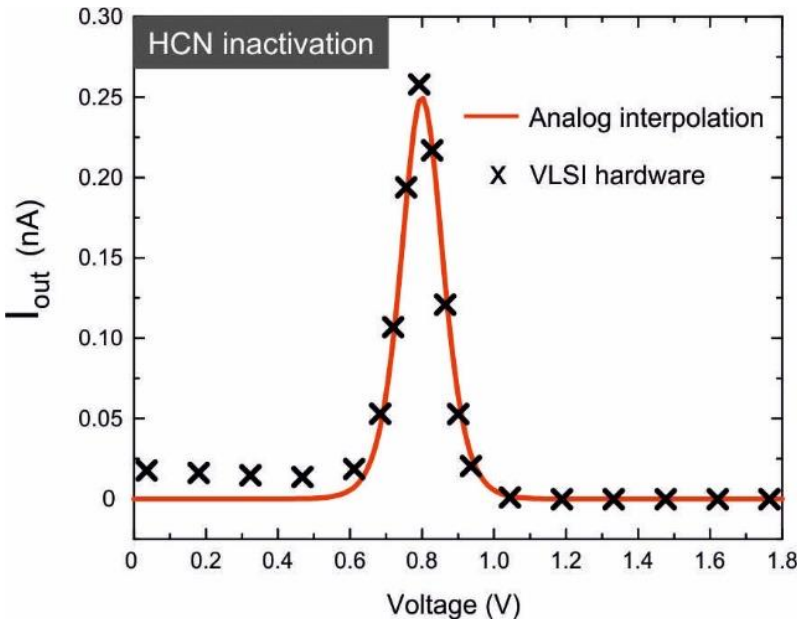

Supplementary Figure 10: Voltage dependence of the gate kinetics of HCN inactivation.
The red line shows the gate kinetics given by Eq.9. The cross symbols show the same kinetics synthesized by the 9-stage circuit of Fig.2(c) as simulated in Spectre®.

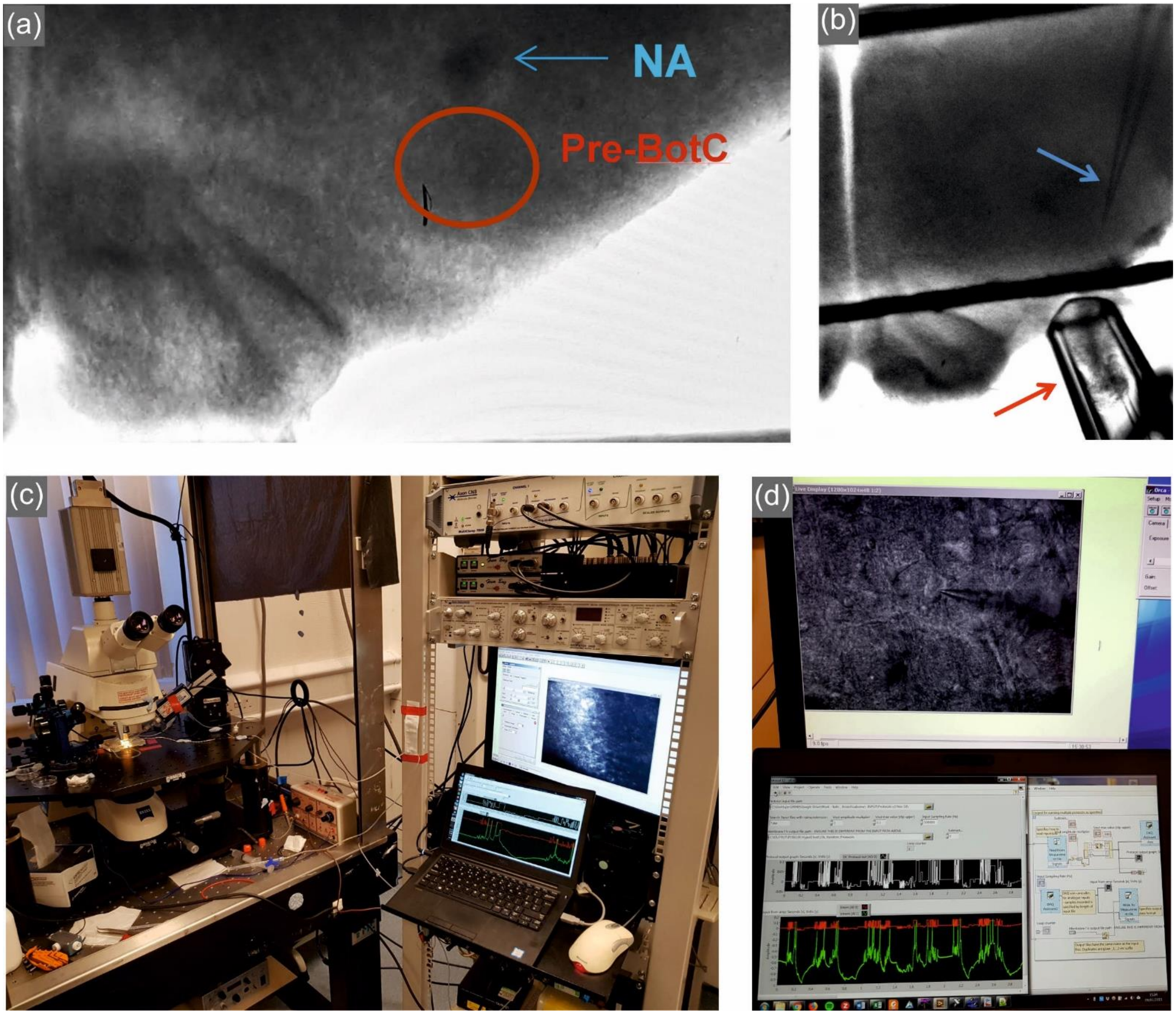

Supplementary Figure 11: Patch clamping of respiratory and pyramidal neurons in rat brain slices.
(a) Slice of the rat brainstem showing the pre-Bötzinger area where the respiratory central pattern generator is located. (b) Suction electrode (red arrow) recording from the XII rootlet whose motor outflow fires in time with the respiratory neuron. Patch pipette targeting the pre-Bötzinger area (blue arrow). (c) Electrophysiological rig. (d) The control panel shows a CCD video of a patched respiratory neuron (top screen). The lower screen shows the current protocol being applied to the respiratory neuron (red trace) and the membrane voltage oscillations being recorded (green trace).

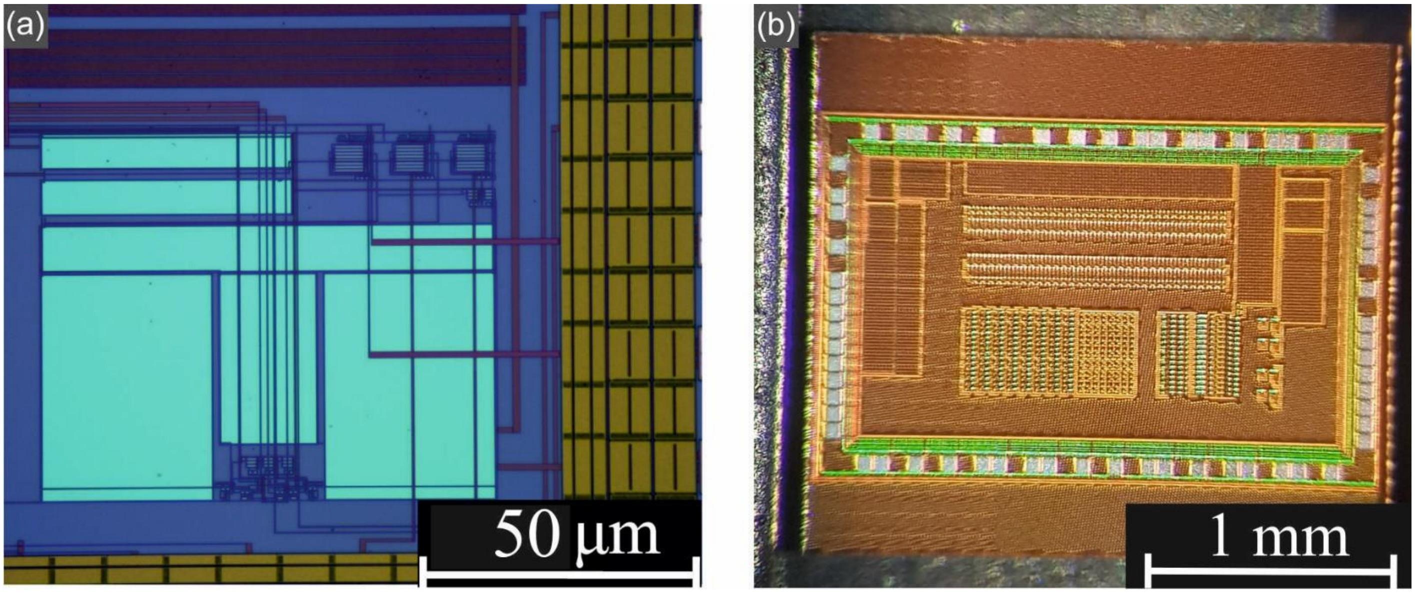

Supplementary Figure 12: VLSI circuit.
(a) VLSI neuron. The larger areas (cyan) are the capacitances C, C_m, C_h ... (b) Overall view of the neuromorphic chip.

II. Supplementary Tables

Ion channel	Parameter ID	Value
	$C_m(\mu\text{F}.cm^{-2})$	1
	$\text{ISA}(cm^2)$	2.9×10^{-4}
Fast and transient Sodium current (NaT)	$g_{NaT}(\text{mS}.cm^{-2})$	69
	$E_{Na}(\text{mV})$	41
	$V_{tm}(\text{mV})$	-39.92
	$\delta V_m(mV^{-1})$	10
	$t_{0m}(\text{mS})$	0.143
	$\epsilon_m(\text{mS})$	1.099
	$\delta V_{\tau m}(mV^{-1})$	23.39
	$V_{th}(\text{mV})$	-65.37
	$\delta V_h(mV^{-1})$	-17.65
	$t_{0h}(\text{mS})$	0.701
	$\epsilon_h(\text{mS})$	12.9
	$\delta V_{\tau h}(mV^{-1})$	27.22
Transient depolarisation activated current (K)	$g_K(\text{mS}.cm^{-2})$	6.9
	$E_K(\text{mV})$	-100
	$V_{tn}(\text{mV})$	-34.58
	$\delta V_{\tau n}(mV^{-1})$	23.39
	$\delta V_n(mV^{-1})$	22.17
	$t_{0n}(\text{mS})$	1.291
	$\epsilon_n(\text{mS})$	4.314
Leak current (L)	$g_L(\text{mS}.cm^{-2})$	0.465
	$E_L(\text{mV})$	-65

Supplementary Table 1. Parameters set in the Hodgkin-Huxley conductance model (Eq.11).
These parameters are taken from a thalamocortical relay neuron[2]**.**

III. Supplementary Notes

Supplementary Note 1: Power consumption of the VLSI neurons (SPECTRE calculations)

Neuron type	Input current	Average power	Peak power	Firing rate
Supplementary Figure 2 neuron	1nA	75.6nW	85.5nW	273 Hz
Resp. neuron	1nA	139nW	187nW	240 Hz

The two neurons are stimulated with 1 nA of DC current. The peak power corresponds to the maximum current the circuit draws as the neuron is firing. The average power is calculated from the average current drawn over a time window of 100ms.

The average power per spike for each neuron is given below. This is compared to a 180 nm integrate-and-fire neuron (Nair, Indiveri, ISCAS 2019).

**Neuron type**	**Energy per spike**
Supplementary Figure 2 neuron	276 pJ per spike
Resp. neuron	579 pJ per spike
180nm Integrate-and-Fire	10 pJ per spike

Supplementary Note 2: Solid state neuron variants

A. Voltage dependent gate recovery times

We found that SSN models often give excellent predictions of biological neuron dynamics if *constant gate recovery times* are used. Supplementary Figure 2 shows a variant of the ionic channel of Fig.1 incorporating voltage-independent gate recovery times.

B. Rasche-Douglas variant implementing activation curves with transconductance amplifiers

An implementation of sigmoidal activation by transconductance amplifiers (Eq.S16) has been proposed by Rasche and Douglas (2000)[1] as an alternative to differential pairs (Eq.S12)[5]. We find that this approach is far less amenable to building quantitative neuron models for two reasons. Unlike the differential pair which outputs a *positive current* (Eq.S12), the current output by the TCA can have *either sign* (Eq.S16). For this reason, TCAs require an extra current mirror placed in output of the TCA to limit the current to positive values. The current mirror introduces a Heaviside function $\theta(x)$ which modifies the SSN model equations as follows:

$$
\begin{aligned}
C\frac{dV}{dt} = {} & I_{gL}\tanh\beta_L\,(E_L - V) + \alpha I_{inj} + I_{dark} \\
& + \{I_{gm}\tanh\beta_m\,(V - V_{tm}) \\
& \quad - I_{gh}\tanh\beta_h\,(V_h - V_{th})\theta(V_h - V_{th})\}\theta(I_m - I_h) \\
& - I_{gn}\tanh\beta_n\,(V_n - V_{tn})\theta(V_n - V_{tn}) \\
C_m\frac{dV_m}{dt} = {} & I_{\tau m}\tanh\beta\,(V - V_m) \\
C_h\frac{dV_h}{dt} = {} & I_{\tau h}\tanh\beta\,(V - V_h) \\
C_n\frac{dV_n}{dt} = {} & I_{\tau n}\,tanh\,\beta\,(V - V_n)
\end{aligned}
\qquad (S1)
$$

The drawbacks of the Rasche-Douglas approach are thus as follows:

- the extra Heaviside functions $\theta(V_h - V_{th})$ and $\theta(V_n - V_{tn})$ in Eq.S1 do not meet the criterion of doubly differentiability required by data assimilation. Data assimilation fails.
- the extra Heaviside functions truncate the (in)activation curves below the (in)activation threshold. Therefore these do not accurately represent biological activation curves.

The SSN model (Eq.10 / Fig.1) is therefore better than Eq.S1 for predicting biological neuron oscillations.

Supplementary Note 3: Adaptation of the CA1 neuron model to current steps

In Supplementary Figure 3, the model predictions (right-hand side) demonstrate the spike frequency adaptation and latency (delay without spiking) of the CA1 neuron to current steps. The same adaptation and latency is observed in CA1 membrane voltage recordings (left-hand side). The model is consistent with the behaviour of CA1 neurons reported by McKiernan and Marrone (2007)[6].

Note that membrane voltage oscillations recorded in response to current steps are known to vary from one epoch to the next where the *same current step is injected*. The poor reliability is ascribed to stochastic resonance [7] and random oscillations of the membrane potential[8]. The need for reliable patch-clamp recordings to test model predictions is a first reason why we use complex current protocols to validate the predictions of completed models. The second reason is that tonic current injection is insufficient to demonstrate the full dynamic range of the model.

Supplementary Note 4: Does the soma of CA1 neuron incorporate calcium channels?

Calcium channels are predominantly located in dendrites. In order to determine whether calcium channels are present in sufficient numbers in the soma to affect its dynamics, we have assimilated the CA1 data of Fig.5 with a model using the AHP calcium activated potassium channel (Supplementary Figure 5) instead of the muscarinic ion channel. The results, shown in Supplementary Figure 4, indicate that although the fits are acceptable (panel a), the extracted parameters take meaningless values with the effect that the predicted membrane voltage misses several action potentials (panels b,c) and the dynamics of gate variables (panels d-f) is wrong. Therefore we can say with a degree of confidence that if Ca channels are present in the soma, their effect on electrical properties is a second order effect.

Bursting vs. non-bursting neurons in the respiratory central pattern generator

The respiratory network has state-dependent modes of operation. These are: eupnoea (normal breathing), apneusis (prolonged inspiration) and gasping. These states reflect reconfigurations of the respiratory network from 3- to 2- to 1-phase neuronal oscillations, respectively. Only in gasping mode is the intrinsic burster neuron deployed[9]. This, however, is irrelevant to our network because our respiratory oscillator requires the 3-phase eupneic network in order to drive the various motor outflows we are studying; one of which is the post-inspiratory control of the vagus nerve to the heart to mediate respiratory sinus arrhythmia. The 3-phase oscillator is driven by different neurones that generate rhythm through reciprocal inhibitory synaptic connections, not intrinsic bursting properties[10].

Supplementary Note 5: First principle analysis of silicon neuron circuits

A. Equations of the differential pair

In Supplementary Figure 6, the drain current (I_D) of a sub-threshold MOSFET increases exponentially with the gate bias (V_g) applied relative to the source (V_s)[3,4]. At saturation ($V_{ds} > 4U_T$) one has:

$$I_D = I_0 \ exp\left(\frac{\kappa V_g - V_s}{U_T}\right), \qquad \text{(S2)}$$

where $U_T \approx 25\ mV$ and $\kappa \approx 0.7$ is the sub-threshold slope factor set by the technology: $\kappa = C_{OX}/(C_{OX} + C_D)$, where C_{OX} is the capacitance of the oxide layer of a MOSFET and C_D is the capacitance of the depletion layer. Using Eq.S2 in the differential pair in Supplementary Figure 6 gives:

$$I_1 = I_0 \ exp\left(\frac{\kappa V - V_s}{U_T}\right), \qquad \text{(S3)}$$

$$I_2 = I_0 \ exp\left(\frac{\kappa V_t - V_s}{U_T}\right). \qquad \text{(S4)}$$

The drain current in the current source transistor (T3) is:

$$\begin{aligned} I_{max} &= I_1 + I_2 \\ &= I_0 \ \ exp\left(\frac{\kappa V - V_s}{U_T}\right) + I_0 \ exp\left(\frac{\kappa V_t - V_s}{U_T}\right) \\ &= I_0 \, exp\left(\frac{-V_s}{U_T}\right)\left[exp\left(\frac{\kappa V}{U_T}\right) + exp\left(\frac{\kappa V_t}{U_T}\right)\right]. \end{aligned} \qquad \text{(S5)}$$

from which one obtains the common source voltage V_s as a function of I_{max} as:

$$exp\left(\frac{-V_s}{U_T}\right) = \frac{I_{max}}{I_0 \ \left[exp\left(\frac{\kappa V}{U_T}\right) + exp\left(\frac{\kappa V_t}{U_T}\right)\right]} \ , \qquad \text{(S6)}$$

Substituting Eq. S6 into Eq. S3 and Eq. S4, one obtains:

$$I_1 = \frac{I_{max} \ exp\left(\frac{\kappa V}{U_T}\right)}{exp\left(\frac{\kappa V}{U_T}\right) + exp\left(\frac{\kappa V_t}{U_T}\right)} \ , \qquad \text{(S7)}$$

and,

(S8)

$$I_2 = \frac{I_{max}\ exp\left(\frac{\kappa V_t}{U_T}\right)}{exp\left(\frac{\kappa V}{U_T}\right) + exp\left(\frac{\kappa V_t}{U_T}\right)}.$$

The output current I_1 effectively takes a sigmoidal form since:

$$I_1 = I_{max}\frac{exp\left(\frac{\kappa V}{U_T}\right)}{exp\left(\frac{\kappa V}{U_T}\right) + exp\left(\frac{\kappa V_t}{U_T}\right)} \times \frac{exp\left(-\frac{\kappa}{U_T}V\right)}{exp\left(-\frac{\kappa}{U_T}V\right)},$$

$$\frac{I_1}{I_{max}} = \frac{1}{1 + exp\left(-\frac{\kappa}{U_T}\ (V - V_t)\right)}. \tag{S9}$$

This expression may be simplified by recalling the $tanh$ function:

$$tanh(x) = \frac{exp(x) - exp(-x)}{exp(x) + exp(-x)}$$

$$= \frac{exp(x)\,(1 - exp(-2x))}{exp(x)(1 + exp(-2x))}$$

$$= \frac{1}{1 + exp(-2x)} - \frac{exp(-2x)}{1 + exp(-2x)}\ , \tag{S10}$$

and setting $u \equiv 2x \equiv \frac{\kappa}{U_T}\ (V - V_t)$:

$$tanh\left(\frac{u}{2}\right) = \frac{1}{1 + exp(-u)} - \frac{exp(-u)}{1 + exp(-u)} \tag{S11}$$

Substituting Eq.S9 into S11 gives:

$$tanh\left(\frac{u}{2}\right) = \frac{I_1}{I_{max}} - \frac{exp(-u) + 1 - 1}{1 + exp(-u)}$$

$$= \frac{I_1}{I_{max}} - 1 - \frac{-1}{1 + exp(-u)}$$

$$= \frac{I_1}{I_{max}} - 1 + \frac{I_1}{I_{max}}$$

$$\frac{I_1}{I_{max}} = \frac{1}{2}\left[1 + tanh\left(\frac{u}{2}\right)\right]$$

$$I_1 = \frac{I_{max}}{2}[1 + tanh\,\beta(V - V_t)] \tag{S12}$$

where we have set $\beta \equiv \frac{\kappa}{2U_T}$. Eq.S12 gives the sigmoidal dependence of (in)activation currents in VLSI circuits. The activation slope β is however a constant in VLSI design whereas it is a parameter in conductance models. Analog interpolation synthesizes an adjustable slope β_m so that the activation current in the SSN model is:

$$I_m = \frac{I_{max}}{2}[1 + tanh\,\beta_m(V - V_{tm})] \tag{S13}$$

B. Equations of the transconductance amplifier

In Supplementary Figure 7, the current output of the TCA, $I_{out} = (I_1 - I_2)$ is given by:

$$I_1 - I_2 = I_{max}\ \frac{exp\left(\frac{\kappa V_t}{U_T}\right) - exp\left(\frac{\kappa V}{U_T}\right)}{exp\left(\frac{\kappa V_t}{U_T}\right) + exp\left(\frac{\kappa V}{U_T}\right)} \tag{S14}$$

$$I_1 - I_2 = I_{max}\ \frac{exp\left(\frac{\kappa V_t}{U_T}\right) - exp\left(\frac{\kappa V}{U_T}\right)}{exp\left(\frac{\kappa V_t}{U_T}\right) + exp\left(\frac{\kappa V}{U_T}\right)} \times \frac{exp\left(-\frac{\kappa}{2U_T}(V_t + V)\right)}{exp\left(-\frac{\kappa}{2U_T}(V_t + V)\right)} \tag{S15}$$

Hence the current output by the TCA is:

$$I_{out} = I_{max}\ tanh\left[\frac{\kappa}{2U_T}(V_t - V)\right] \tag{S16}$$

C. Equations of the leak current

In Supplementary Figure 8, the *leak current* is given by:

$$I_{Leak} = I_{gl}\ tanh\left[\frac{\kappa}{2U_T}(E_L - V)\right] \tag{S17}$$

D. Equations of the after-hyperpolarization current (AHP)

A circuit modelling the AHP current has been proposed by Rasche and Douglas (2000)[1]. We give here the equations derived from this circuit which we used to construct SSN models incorporating the AHP current. Although we used the AHP current to analyse hippocampal and respiratory neurons, the AHP channel was found to have negligible conductance in the respiratory neuron. In the hippocampal neuron, the M-current always produced better fits of the membrane voltage than the AHP current (see Fig.5 and Supplementary Figure 4). Hence the AHP channel could be omitted from Table III.

In Supplementary Figure 5, an action potential ($V > V_{Pth}$) increases the intracellular calcium concentration by triggering an influx of calcium ions into the soma (I_{Cain}). This concentration then decays slowly due to calcium buffering. The intracellular calcium concentration is modelled by gate variable V_c which has first order dynamics given by:

$$C_c \frac{dV_c}{dt} = I_{\tau c} \, tanh[\, \beta(V_{carest} - V_c)] + I_{cain} \, \theta[V - V_{PTH}] \tag{S18}$$

Above a threshold concentration ($V_c > V_{tc}$) the calcium ions activate the following influx of potassium ions:

$$I_{AHP} = I_{gc} \, tanh[\beta(V_c - V_{tc})]\theta[V_c - V_{tc}] \tag{S19}$$

Where $\theta(x)$ is the Heaviside step function.

Supplementary References

1. Rasche, C. & Douglas, R. An Improved Silicon Neuron. *Analog Integr. Circuits Signal Process.* **23**, 227–236 (2000).
2. McCormick, D. A. & Huguenard, J. R. A model of the electrophysiological properties of thalamocortical relay neurons. *J. Neurophysiol.* **68**, 1384–1400 (1992).
3. Mead, C. *Analog VLSI and Neural. Addison-Wesley Longman Publishing Co.,Inc.* (Addison-Wesley, 1989).
4. Liu, S.-C., Delbruck, T., Kramer, J., Indiveri, G. & Douglas, R. *Analog VLSI : circuits and principles*. (MIT Press, 2002).
5. Mahowald, M. & Douglas, R. A silicon neuron. *Nature* **354**, 515–518 (1991).
6. McKiernan, E. C. & Marrone, D. F. CA1 pyramidal cells have diverse biophysical properties, affected by development, experience, and aging. *PeerJ* **5**, e3836 (2017).
7. Tateno, T., Harsch, A. & Robinson, H. P. C. Threshold Firing Frequency–Current Relationships of Neurons in Rat Somatosensory Cortex: Type 1 and Type 2 Dynamics. *J. Neurophysiol.* **92**, 2283–2294 (2004).
8. Long, M. A., Jin, D. Z. & Fee, M. S. Support for a synaptic chain model of neuronal sequence generation. *Nature* **468**, 394–399 (2010).
9. Paton, J. F. R., Abdala, A. P. L., Koizumi, H., Smith, J. C. & St-John, W. M. Respiratory rhythm generation during gasping depends on persistent sodium current. *Nat. Neurosci.* **9**, 311–313 (2006).
10. Smith, J. C., Abdala, A. P. L., Koizumi, H., Rybak, I. A. & Paton, J. F. R. Spatial and Functional Architecture of the Mammalian Brain Stem Respiratory Network: A Hierarchy of Three Oscillatory Mechanisms. *J. Neurophysiol.* **98**, 3370–3387 (2007).
